# High‐Calorie Diet During Pregnancy Leads to Muscular Fibrosis and Neuromuscular Damage in Offspring Mice

**DOI:** 10.1002/jcsm.70027

**Published:** 2025-09-16

**Authors:** Jun Seok Son, Song Ah Chae, Yoon Ha Chun, Hongyang Wang, Zhihua Jiang, Min Du

**Affiliations:** ^1^ Nutrigenomics and Exercise Biology Laboratory, Department of Obstetrics, Gynecology & Reproductive Sciences University of Maryland School of Medicine Baltimore Maryland USA; ^2^ Department of Physiology University of Maryland School of Medicine Baltimore Maryland USA; ^3^ Nutrigenomics and Growth Biology Laboratory, Department of Animal Sciences Washington State University Pullman Washington USA; ^4^ Cell Biology Group, Department of Surgery University of Maryland School of Medicine Baltimore Maryland USA; ^5^ Institute of Animal Husbandry and Veterinary Science Shanghai Academy of Agriculture Sciences Shanghai China; ^6^ Department of Animal Sciences Washington State University Pullman Washington USA

**Keywords:** aging, maternal high‐fat diet, mitochondria, pregnancy, sarcopenia, skeletal muscle

## Abstract

**Background:**

Sarcopenia, recognized as an age‐related loss of muscle mass and function, is a critical risk for geriatric health. We previously demonstrated that maternal high‐fat diet (HFD) suppresses mitochondrial biogenesis during fetal skeletal muscle development, but the longitudinal effect of maternal HFD challenge on offspring muscle sarcopenia and fitness impairment remains unclear. Mitochondrial polymerase γ (PolG) mutation accelerates mitochondrial DNA mutations and leads to premature aging.

**Methods:**

To determine the mechanisms underlying the longitudinal effect of maternal HFD challenge on offspring sarcopenia and aging, heterozygote mitochondrial polymerase γ mutated (*PolgA* ^mut/+^) female mice were fed either a control diet (CD) or HFD during pregnancy, which were mated with heterozygote *PolgA* male mice. Thus, we had four experimental groups: maternal CD (M‐CD) + WT, M‐CD + *PolgA* ^mut^, M‐HFD + WT and M‐HFD *+ PolgA* ^mut^. Six‐month‐old offspring mice were utilized for testing metabolic health, maximal muscle strength and cardiorespiratory fitness capacity. Then, 9‐month‐old offspring mice were used for biochemical and histochemical analyses.

**Results:**

Maternal high‐calorie diet during pregnancy decreased offspring muscle strength and cardiorespiratory function (*p* < 0.05), which were associated with loss of muscle mass (*p* < 0.05). These adverse outcomes were most dramatic in M‐HFD with PolG mutation (*p* < 0.05). Maternal HFD challenge activated muscle atrophy signalling, including MuRF1 and Atrogin‐1 (*p* < 0.05), which were worsened in *PolgA* mice (*p* < 0.05). Furthermore, M‐HFD increased the accumulation of intramuscular fibrosis in *PolgA* offspring (*p* < 0.05). In addition, M‐HFD increased the risk of neuromuscular damage by attenuating GABA_A_ receptor pathway in *PolgA* mice (*p* < 0.05).

**Conclusions:**

Maternal high‐calorie diet during pregnancy induced offspring muscle atrophy and intramuscular fibrosis, especially with PolG mutation, underscoring mitochondrial dysfunction in linking maternal HFD to offspring premature aging.

## 1 Introduction

The global population of elderly adults over the age of 65 is growing fast. Furthermore, the obesity rate in this elderly population has been increasing [1]. Sarcopenia, known as the loss of muscle mass, strength and function, occurs during aging [2]. In addition, the percentage of body fat increases up to the seventh decade of life and then decreases [3]. Increased fat mass negatively affects life span and accelerates skeletal muscle sarcopenia [4, 5].

Together with the overall population, obesity in women during the reproductive age is also increasing, accounting for 42% in the US. Maternal obesity (MO) predisposes fetal macrosomia [6] and offspring metabolic dysfunction [7, 8], which is closely associated with mitochondrial dysfunction in skeletal muscle [9, 10]. Constantly, a maternal high‐fat diet (HFD), which is prevalent in western societies, during pregnancy results in metabolic abnormality in offspring [11]. Our previous study showed that maternal HFD‐induced metabolic dysfunction impedes mitochondrial biogenesis and muscle development in the offspring [10]. HFD during pregnancy induces mitochondrial dysfunction, suppresses fatty acid oxidation and elicits insulin resistance in offspring skeletal muscle [12, 13].

Proper mitochondrial function is indispensable for the metabolic health of muscle and other tissues and organs. In addition, mitochondria are a major driver of cell lineage commitment and differentiation, and its dysfunction induces fibrotic diseases [14]. Moreover, excessive intramyocellular lipid accumulation is correlated with lipotoxicity, contributing to fibrotic development [15]. During aging, cellular plasticity and muscle regeneration capacity decline, leading to the progressive replacement of intramuscular fibrotic and fatty tissues [16, 17], impeding muscle strength and function [18, 19]. Accumulating studies showed that MO and HFD induce fibrosis in the skeletal muscle of offspring, which correlates with metabolic dysfunction [20, 21, 22, 23, 24, 25, 26]. However, their effects on the metabolic dysfunction of offspring skeletal muscle during aging and the risk factors of sarcopenic obesity remain to be tested.

To examine the roles of mitochondrial dysfunction in linking maternal HFD to offspring metabolic dysfunction, we used mitochondrial polymerase γ mutated *PolgA* ^D257A^ mice. In these mice, the proofreading function of PolG is abolished, accelerating mitochondrial DNA (mtDNA) mutations and aging, with a lifespan around 13–15 months versus wildtype mice around 28 months [27, 28]. This mutant mouse model allowed us to analyse the mediatory role of mitochondrial mutagenesis and dysfunction in linking maternal HFD to offspring skeletal muscle aging. Using these mice, we hypothesized that maternal HFD accelerates skeletal muscle fibrosis and metabolic dysfunction in offspring, which is worsened in *PolgA* offspring, showing that mitochondrial dysfunction is a key factor impairing muscle function of offspring born to HFD mothers.

## 2 Materials and Methods

### 2.1 Animal Procedures

All animal experiments were performed in AAALAC‐approved facilities according to protocols approved by the Institutional Animal Care and Use Committee (IACUC) at the University of Maryland School of Medicine (#AUP‐00000151) and Washington State University (#6704). Homozygous DNA polymerase γ gene (gene: *Polg*, protein: PolG) mutated mice (*PolgA* ^D257A^, #017341) were purchased from the Jackson Laboratory. Ten‐ to 12‐week‐old female heterozygous *PolgA* ^D257A^ mutated mice were randomized into two groups: a control diet (CD, *n* = 6) or an HFD (*n* = 6), and fed either a CD (10% energy from fat, #D12450J, Research Diets, New Brunswick, NJ) or an HFD (45% energy from fat, #D12451, Research Diets) 1 week before mating with age‐matched male heterozygous *PolgA* ^D257A^ mutated mice fed a chow diet. Mating was determined by the presence of vaginal smears.

After birth, all maternal diets were replaced on the chow diet, and the offspring mice continued to feed on the chow diet until 9 months old. Then, these 9‐month‐old offspring mice were anaesthetised following 5 h fasting for additional analyses. Given that oestrogen plays a critical role in enhancing muscle mass and strength in women [29, 30] and its decline during aging might confound data interpretation, we only utilized and showed data of male offspring.

### 2.2 Indirect Calorimetry

Indirect open‐circuit calorimetry was conducted by using Comprehensive Lab Animal Monitoring System (Columbus Instruments, Columbus, OH). Six‐month‐old offspring mice were acclimated to metabolic cages for 1 h, and then 24 h indirect calorimetry including oxygen consumption rates (OCRs), carbon dioxide production rates, and respiratory exchange ratio (RER) was recorded, as described in our previous studies [6, 31]. Offspring mice were fed ad libitum with the chow diet and fresh water during measurement.

### 2.3 Endurance Treadmill Exercise Capacity Test

Six‐month‐old offspring mice were subjected to an adaptation for treadmill exercise with an intensity (speed) of 10 m/min for 10 min, three times within 1 week. Endurance treadmill exercise capacity test was determined using a 25° inclined treadmill exercise along with the indirect respiratory metabolic measurements using a treadmill respiratory measurement system (Oxymax fast 4 lane modular treadmill system; Columbus Instruments) according to the manufacturer's protocols and guidelines, as described in our previous studies [9, 32].

### 2.4 Forelimb Grip Strength Test

The forelimb grip strength of 6‐month‐old offspring mice was measured using a grip strength metre (Columbus Instruments), as described in our previous studies [6]. Briefly, the repetition maximum (RM) was recorded during 10 continuous measurements, which were utilized to determine maximal forelimb grip strength and endurance grip strength as described in our previous studies [6, 9, 10]. Data collection was performed in accordance with a single‐blind test.

### 2.5 Histological Analysis

Offspring mice were euthanized, and tibialis anterior (TA) muscles were incubated for 48 h in 4% paraformaldehyde (PFA), transferred to 70% ethanol, and then these samples were embedded and sectioned (5‐μm‐thickness) for Masson's trichrome and haematoxylin and eosin (H&E) staining. Images were acquired on the EVOS XL Core Imaging System (Thermo Fisher Scientific, Waltham, MA, USA). For muscle fibre type distribution analysis, fresh TA muscles were embedded in OCT compound (Fisher Scientific, Waltham, MA, USA) and sectioned (5‐μm‐thickness) for immunocytochemical (ICC) staining. The primary antibodies, including anti‐myosin heavy chain (MHC) I (#BA‐F8), anti‐MHC IIa (#SC‐71) and anti‐MHC IIb (#BF‐F3) mouse monoclonal antibodies, were purchased from the Developmental Studies Hybridoma Bank (Iowa City, IA, USA). Anti‐Col1a mouse monoclonal antibody was purchased from Santa Cruz Biotechnology (Dallas, TX, USA). For the secondary antibodies, goat anti‐mouse IgG2b Alexa 488, goat anti‐mouse IgG1 Alexa 555 and goat anti‐mouse IgM Alexa 350 antibodies were purchased from Thermo Fisher Scientific. Images were captured by a Nikon Eclipse Ni microscope (Nikon Instrument Inc., NY, USA).

### 2.6 RNA‐seq and Bioinformatic Analyses

The gastrocnemius muscle was collected from 9‐month‐old offspring. Total RNA was prepared using TRIzol reagent followed by the manufacturer's instructions (Invitrogen, Grand Island, NY) and used for the next‐generation whole transcriptome termini site sequencing, as described in our previous studies [9, 10, 31].

### 2.7 Chromatin Immunoprecipitation qPCR (ChIP‐qPCR) Assay

ChIP‐qPCR assays were performed as previously described [33]. Briefly, homogenized gastrocnemius muscles were cross‐linked with 1% formaldehyde, resuspended in PBS with glycine and lysed in a ChIP lysis buffer (10 mmol/L Tris–HCl pH = 8.0, 10 mmol/L NaCl, 1% SDS, 3 mmol/L MgCl_2_, 0.5% NP‐40) with protease inhibitor cocktail (Roche, Millipore Sigma, Burlington, MA, USA). After sonication, the supernatant was precleaned with ChIP‐grade Pierce magnetic protein A/G beads (Thermo Fisher Scientific). Then, these were incubated with H3K4me3 (#9751, Cell Signaling Technology, Danvers, MA, USA) or rabbit IgG antibody (#30000‐0‐AP, Proteintech, Rosemont, IL, USA), precipitated with magnetic beads and treated with RNaseA and proteinase K (#25530, Thermo Fisher Scientific). For qPCR analysis, the samples were utilized using PowerUp SYBR Green Master Mix (Applied Biosystems, Waltham, MA, USA). IgG was used for normalization for enrichment folds. Primer sequences were *Pgc1a*‐ChIP‐A forward: 5′‐CAGGAGATTTGAGTTATTATGTGAGCA‐3′; reverse: 5′‐TGAAGTAACGCTTAGAGAGAGAGGAA‐3′; *Pgc1a*‐ChIP‐B forward: 5′‐TTCCTCTCTCTCTAAGCGTTACTTCA‐3′; reverse: 5′‐CTTACTACAGTCCCCAGTCACATGA‐3′.

### 2.8 Immunoblotting

Protein was extracted from gastrocnemius muscle of 9‐month‐old offspring using lysis buffer (10 mM Tris–HCl, 150 mM NaCl, 0.5% Triton X‐100, 1.0 mM EDTA, 10% Glycerol, 1.0 mM NaF, 1.0 mM Na_3_VO_4_, 1 mM DTT) with Protease Inhibitor Cocktail (Roche cOmplete, Mannheim, Germany). Protein lysate concentrations were determined by BCA Protein Assay Kit II (BioVision, Milpitas, CA, USA). The primary antibodies were used: p‐Akt^Thr308^ (#29163‐1‐AP; RRID:AB_2918241), Akt (#10176‐2‐AP; RRID:AB_2224574), p‐mTOR^Ser2448^ (#67778‐1‐IG; RRID:AB_2889842), mTOR (#66888‐1‐IG; RRID:AB_2882219), p‐P70S6K^Thr389^ (#28735‐1‐AP; RRID:AB_2918197), P70S6K (#14485‐1‐AP; RRID:AB_2269787), FNDC5 (#23995‐1‐AP; RRID:AB_2879394), BDNF (#28205‐1‐AP; RRID:AB_2818984), GDF11 (#26715‐1‐AP; RRID:AB_2918107), FASN (#10624‐2‐AP; RRID:AB_2100801), PGC‐1α (#66369‐1‐IG; RRID:AB_2828002), GAPDH (#10494‐1‐AP; RRID:AB_2263076) and β‐tubulin (#66240‐1‐IG; RRID:AB_2881629) were purchased from Proteintech (Rosemont, IL, USA). OXPHOS (#45‐8099; RRID:AB_2533835), APLN (#PA5‐114860; RRID:AB_2899496) and PRDM16 (#PA5‐20872; RRID:AB_11154178) were purchased from Invitrogen (Rockford, IL, USA). SPARC (#8725; RRID:AB_10860770) and VDAC (#4661; RRID:AB_10557420) were also purchased from Cell Signaling Technology (Danvers, MA, USA). Antibodies against GABA_A_Rα1‐6 (#sc‐376282; RRID:AB_10988210), Col1a (#sc‐59772; RRID:AB_1121787), MuRF1 (#sc‐398608; RRID:AB_2819249) and Atrogin‐1 (#sc‐166806; RRID:AB_2246982) were purchased from Santa Cruz Biotechnology. SREBP‐1 antibody was purchased from BD Biosciences (#557036; RRID:AB_396559; Franklin Lakes, NJ, USA). For secondary antibodies used for the detection of target proteins, IRDye 680RD goat anti‐mouse and IRDye 800CW goat anti‐rabbit secondary antibodies were purchased from LI‐COR Biosciences (Lincoln, NE, USA). The membranes were stripped by ReBlot Plus Mild Antibody Stripping Solution (Millipore, St. Louis, MO, USA) for further utilization if needed. The target proteins were detected by ChemiDoc MP Imaging System (Bio‐Rad) as previously described [34].

### 2.9 Statistics

Statistical analyses were based on a two‐way ANOVA followed by Student's *t*‐test, which were performed using SPSS Statistics Ver. 21 (IBM Corp., Armonk, NY, USA) and visualized using GraphPad Prism Ver. 9 (GraphPad Software, San Diego, CA, USA). Data are representative of mean ± SEM. RNA‐seq data were analysed using R Statistics and SPSS Statistics for differentially expressed genes (DEGs), gene ontology (GO) and hierarchical cluster analysis. The number of samples and each dot representing one litter are indicated in the figures and respective legends. *p* values less than 0.05 were considered statistically significant. Statistical differences were indicated as follows: *,#*p* < 0.05, **,##*p* < 0.01 and ***,###*p* < 0.001.

## 3 Results

### 3.1 Maternal HFD Impedes Metabolism and Muscle Function in Aged Offspring

**FIGURE 1:**
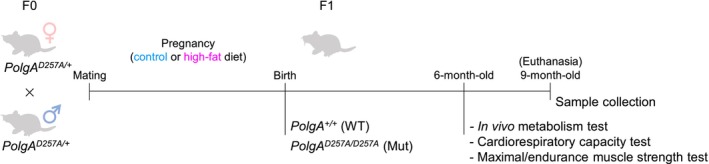
Overview of the study design including *PolgA* ^D257A^ mutant mice and maternal HFD challenge during pregnancy and offspring metabolic and fitness tests.

**FIGURE 2:**
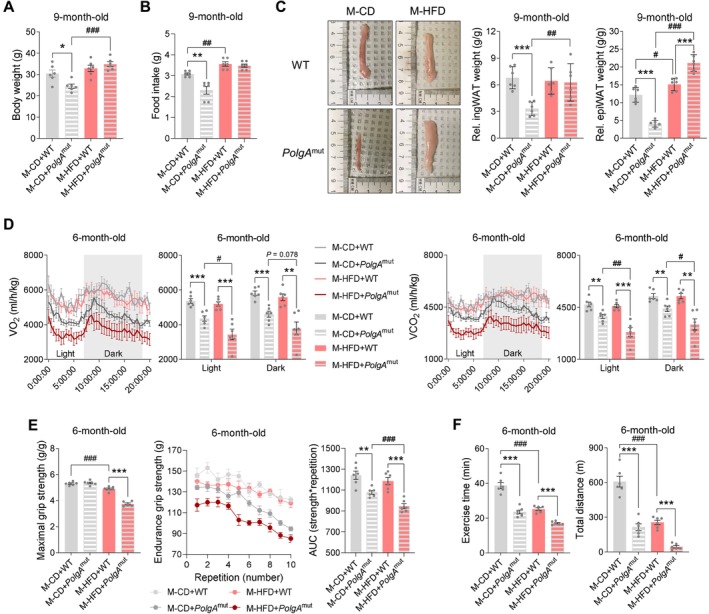
Maternal high‐calorie diet markedly alters muscle fitness function in *PolgA* offspring mice. (A and B) Body weight (A) and calorie intake (B) in *PolgA* offspring with/without M‐HFD challenge during pregnancy (*n* = 6/group). (C) Relative inguinal white adipose tissue (ingWAT) and epididymal (epi)WAT weights in *PolgA* offspring (body weight was used for normalization; *n* = 6/group). (D) Time‐resolved oxygen consumption (left) and carbon dioxide production (right) of 6‐month‐old *PolgA* offspring born from mothers fed HFD during pregnancy (*n* = 6/group). (E) Relative maximal grip strength (body weight was used for normalization) (left) and 10‐repitition endurance grip strength (middle) and area under the curve (AUC) of endurance strength (right) in 6‐month‐old *PolgA* offspring with/without M‐HFD challenge (*n* = 6/group). (F) Total exercise time (left) and distance (right) through cardiorespiratory exercise test in 6‐month‐old *PolgA* offspring in response to M‐HFD (*n* = 6/group). Mean ± SEM, and each dot represents one litter. *p < 0.05, **p < 0.01 and ***p < 0.001 in WT vs. *PolgA* ^mut^, and #*p* < 0.05, ##*p* < 0.01 and ###*p* < 0.001 in CD vs. HFD by two‐sided *p* values by two‐way ANOVA followed by Tukey's test (A–F).

We have shown that maternal metabolic health can intergenerationally regulate mitochondrial activity in offspring skeletal muscle [9, 10]. To determine whether maternal HFD‐induced metabolic dysfunction affects mitochondria‐induced muscle aging, we utilized heterozygous *PolgA* mice to generate WT and mutant offspring mice (Figure 1). We measured basic characteristics including body weight, food intake and weight of adipose tissues (ATs) in 9‐month‐old offspring, with maternal HFD offspring increasing fat accumulation (Figure 2A–C). Because 9‐month‐old experimental *PolgA* ^D257A^ mutated mice display an aging phenotype [28], fitness tests and metabolic analysis were conducted at 6 months old to avoid negative impacts due to premature aging. To pinpoint metabolic phenotypes in the offspring, we performed in vivo metabolic analysis of 6‐month‐old offspring mice using indirect open‐circuit calorimetry. Oxygen consumption rates (OCRs) and carbon dioxide production rates markedly decreased in M‐HFD offspring with PolG mutation, so for respiratory exchange ratios (RERs) (Figures 2D and S1A). The fat oxidation was decreased in CD mice due to PolG mutation, but CHO oxidation was decreased the most profoundly in M‐HFD *PolgA* offspring (Figure S1B,C). To further examine the fitness capacity of offspring mice, we tested grip strength and cardio‐respiratory endurance capacity in 6‐month‐old offspring. M‐HFD offspring showed dramatic decreases in maximal grip strength both in WT and *PolgA*, but *PolgA* itself did not alter the strength (Figure 2E). Consistently, the endurance strength became weaker due to M‐HFD in *PolgA* mice (Figure 2E). We performed VO_2_max analysis for determining cardio‐respiratory fitness capacity [35] in 6‐month‐old offspring mice and found that M‐HFD dramatically reduced total exercise time and distance with *PolgA* (Figure 2F), consistent with the reduction of grip strength.

### 3.2 Maternal HFD and *PolgA* During Pregnancy Alter Offspring Muscle Structure and Fibre Composition

**FIGURE 3:**
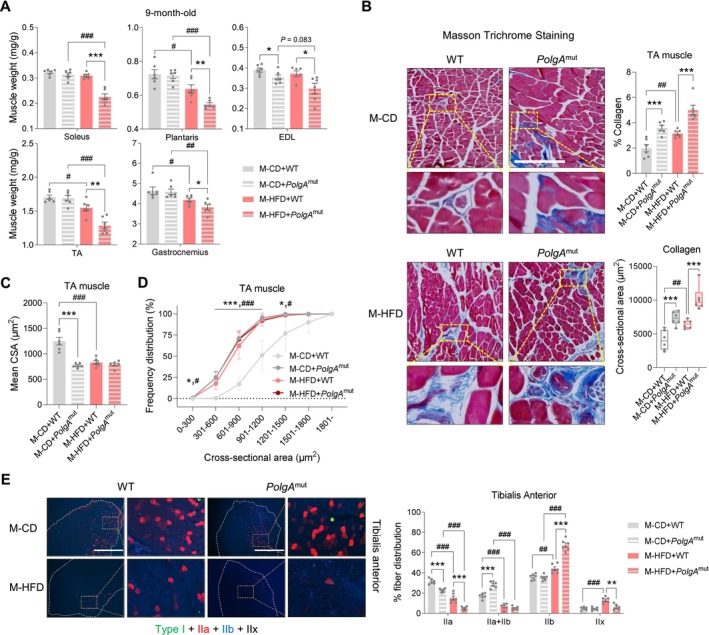
Maternal high‐fat diet during pregnancy changes skeletal muscle structure and fibre types in *PolgA* offspring. (A) Weights of several types of hindlimb muscle including *soleus*, *plantaris*, *extensor digitorum longu*s (EDL), *tibialis anterior* (TA), *gastrocnemius* in 9‐month‐old *PolgA* offspring with/without M‐HFD challenge (*n* = 6/group). (B) Representative images of Masson trichrome staining in 9‐month‐old *PolgA* offspring TA muscle in response to M‐HFD challenge, scale bars represent 200 μm. (C and D) Mean cross‐sectional areas (CSAs) (C) and percent frequency distribution (D) of muscle fibres in *PolgA* offspring TA muscle in response to M‐HFD challenge (*n* = 6/group). (E) Representative images of immunocytochemical (ICC) staining (left) and percent fibre distributions (right) for myosin chain types in 9‐month‐old *PolgA* offspring muscle after M‐HFD challenge, scale bars represent 1000 μm (*n* = 6/group). Mean ± SEM, and each dot represents one litter. *p < 0.05, **p < 0.01 and ***p < 0.001 in WT vs. *PolgA* ^mut^, and #*p* < 0.05, ##*p* < 0.01 and ###*p* < 0.001 in CD vs. HFD by two‐sided *p* values by two‐way ANOVA followed by Tukey's test (A–E).

Interestingly, M‐HFD challenge induced severe muscle loss and fat accumulation, especially in *PolgA* mice (Figures 2C and 3A), leading to obesity with depleted muscle mass termed as sarcopenic obesity (sarcobesity) [36, 37, 38]. Intramuscular fibrosis is one of the major histopathological changes due to aging‐associated muscle loss, sarcopenia [16, 39, 40]. We further performed Masson trichrome staining for examining muscular interstitial fibrosis/collagen accumulation and found that M‐HFD increased the percentage and cross‐sectional area (CSA) of interstitial collagen in offspring TA muscle (Figure 3B). Of note, M‐HFD highly increased collagen content in *PolgA* offspring muscle (Figure 3D). In addition, M‐HFD reduced the mean CSAs of muscle fibres and increased the percentage of small muscle fibres in offspring TA muscle (Figure 3C,D). In addition, M‐HFD reduced the percentage of type IIa fibres (high density mitochondria with high oxidative capacity) (Supporting Information S1: S1), but increased the percentage of type IIb fibres (anaerobic fibres with low mitochondria density) (Supporting Information S1: S1) (Figure 3E). Collectively, our key findings suggest that maternal HFD is deleterious to muscle structure, worsened by the presence of *PolgA* in offspring mice.

**FIGURE 4:**
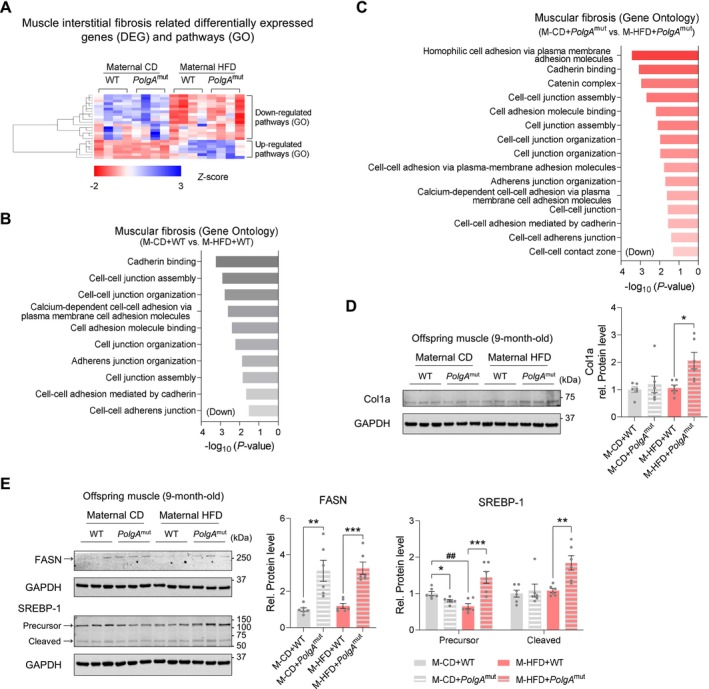
Maternal HFD challenge with *PolgA* exhibits intramuscular fibrosis and fat accumulation. (A and B) Heat map (A) and GO analysis (B) of muscle fibrosis‐related DEGs in 9‐month‐old offspring muscle with/without M‐HFD (*n* = 4/group). (C) GO pathway of muscular fibrosis DEGs in M‐HFD offspring in a subset of *PolgA* ^mut^ (*n* = 4/group). (D) Cropped western blots of Col1a protein levels in either 9‐month‐old WT and *PolgA* offspring muscle in response to maternal HFD challenge during pregnancy (GAPDH was used for normalization; *n* = 6/group). (E) Cropped western blot images of FASN and SREBP‐1 precursor and cleaved protein levels in *PolgA* offspring muscle in response to M‐HFD (GAPDH was used for normalization; *n* = 6/group). Mean ± SEM, and each dot represents one litter. *p < 0.05, **p < 0.01 and ***p < 0.001 in WT vs. *PolgA* ^mut^, and ##*p* < 0.01 in CD vs. HFD by two‐sided *p* values by two‐way ANOVA followed by Tukey's test (D, E).

To further analyse related changes, we performed RNA‐seq analysis using the gastrocnemius muscle of offspring at 9 months of age, showing an increased intramuscular fibrotic response including the decreases of cell–cell junction assembly and adhesion‐related gene expression in offspring muscle of M‐HFD (Figure 4A,B), consistent with M‐HFD with *PolgA* offspring (Figure 4C). In agreement, the levels of collagen type I alpha (Col1a) were not altered by either *PolgA* or M‐HFD alone, but M‐HFD with *PolgA* had a synergistic effect in elevating collagen levels (Figure 4D). Aging also induced fat accumulation in skeletal muscle, which has negative relationships with muscle mass and strength (Supporting Information S1: S2). Indeed, the protein levels of fatty acid synthase (FASN) and sterol regulatory element‐binding protein 1 (SREBP‐1) were elevated in M‐HFD *PolgA* offspring (Figure 4E). Together, these data show that M‐HFD challenge increases intramuscular fibrosis and fat accumulation in aged offspring muscle.

### 3.3 Maternal HFD During Pregnancy Impairs Anabolic Pathways and Induces Muscle Atrophy in Offspring Muscle

**FIGURE 5:**
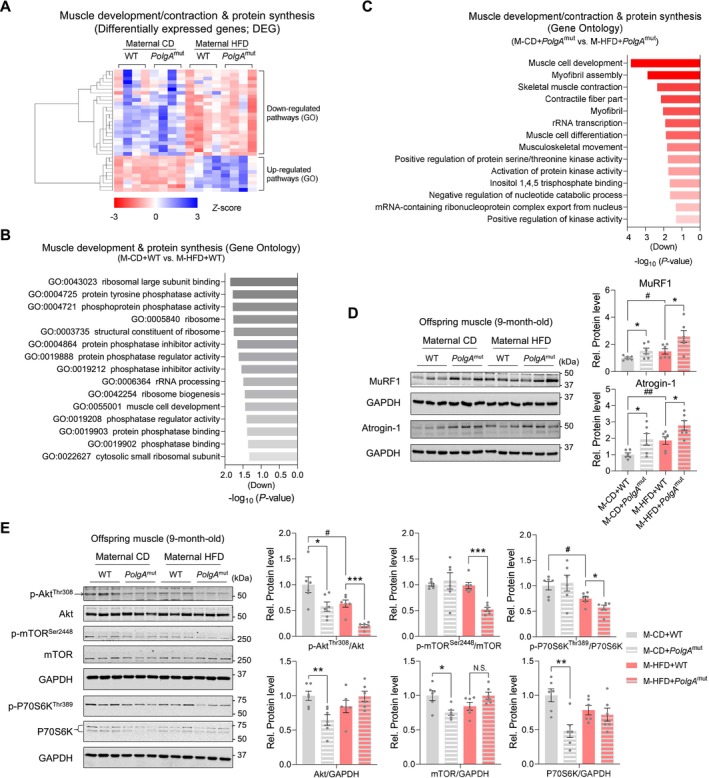
Maternal HFD challenge induces muscle atrophy signalling in *PolgA* offspring mice. (A–C) Expression profile by heat map analysis (A) and GO pathways (B, C) of differentially expressed genes related to muscle development and contraction and protein synthesis (*n* = 4/group). (D) Cropped western blot images of MuRF1 and Atrogin‐1 in *PolgA* offspring muscle in response to M‐HFD (GAPDH was used for normalization; *n* = 6/group). (E) Cropped western blots of phosphorylated Akt (Thr308), phosphorylated mTOR (Ser2448) and phosphorylated P70S6K (Thr 389) protein levels in 9‐month‐old *PolgA* offspring muscle in response to maternal HFD challenge during pregnancy (GAPDH or each total protein level was used for normalization; *n* = 6/group). Mean ± SEM, and each dot represents one litter. *p < 0.05, **p < 0.01 and ***p < 0.001 in WT vs. *PolgA* ^mut^, and #*p* < 0.05 and ##*p* < 0.01 in CD vs. HFD by two‐sided *p* values by two‐way ANOVA followed by Tukey's test (D, E).

To define the underlying molecular signalling pathways that predisposed sarcobesity in offspring mice born to M‐HFD, we analysed RNA‐seq data and found the deterioration of muscle development and protein synthesis GO pathways in M‐HFD (Figure 5A,B), which was more severely impaired in *PolgA* mice (Figure 5C). The muscle anabolic signalling mediators, phosphorylation of Akt^Thr308^ mammalian target of rapamycin (mTOR)^Ser2448^ and P70S6K^Thr389^ were reduced depending on M‐HFD, especially in M‐HFD with *PolgA* (Figure 5E). Meanwhile, the protein levels of muscle RING finger protein 1 (MuRF1), an E3 ubiquitin ligase responsible for muscle remodelling (Supporting Information S1: S3 and S4), and Atrogin‐1, a muscle‐specific F‐box protein (MAFbx1) closely associated with muscle atrophy (Supporting Information S1: S3), were elevated in M‐HFD offspring mice (Figure 5D). In short, our data show that maternal HFD during pregnancy and *PolgA* impair muscle anabolism in aged offspring muscle, leading to muscular atrophy.

The levels of exercise‐induced cytokines termed exerkines in the skeletal muscle are positively associated with physical performance and cardio‐respiratory fitness (Supporting Information S1: S5 and S6). RNA‐seq data generated from 9‐month‐old offspring muscle showed a reduction in exerkine expression in response to M‐HFD (Figure S2A), further reduced because of *PolgA* (Figure S2B). Consistently, the contents of representative exerkines, including fibronectin type III domain containing 5 (FNDC5)/irisin, apelin (APLN), brain‐derived neurotrophic factor (BDNF), growth differentiation factor 11 (GDF11), PR domain containing 16 (PRDM16) and secreted protein acidic and cysteine rich (SPARC) (Supporting Information S1: S5 and S7) were drastically downregulated because of M‐HFD in *PolgA* offspring (Figure S3A,B). We also found positive correlations between exerkines and physical fitness, including maximal grip strength and total exercise time, and exerkines (Figure S3C). Taken together, our data suggest that physical performance and its associated exerkines are deteriorated by maternal HFD during pregnancy in offspring mice which were worsened because of *PolgA*, hinting at the mediatory roles of mitochondria in impairing offspring muscle properties due to M‐HFD.

### 3.4 HFD Challenge During Pregnancy Induces Mitochondrial Metabolic Dysfunction in 9‐Month‐Old Offspring Muscle

**FIGURE 6:**
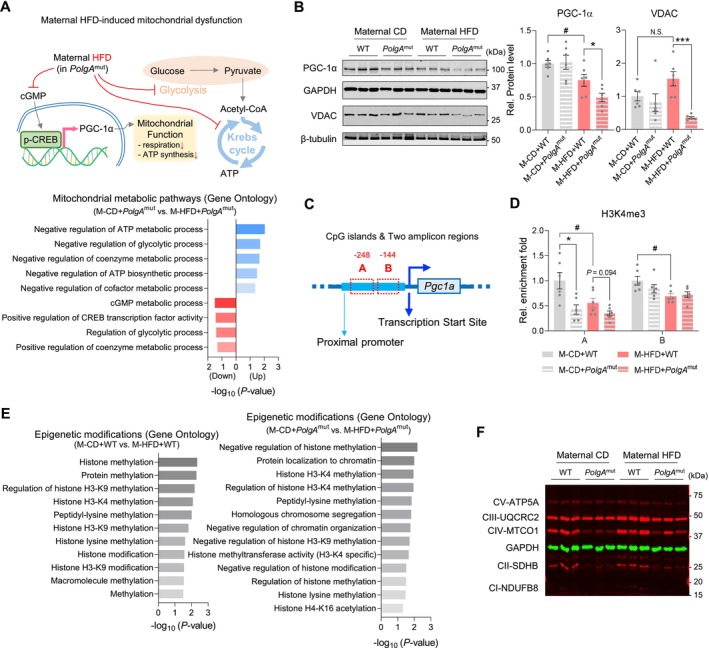
Maternal HFD challenge impedes metabolic phenotypes in *PolgA* offspring. (A) Schematic diagram based on GO analysis (*n* = 4/group): M‐HFD impedes PGC‐1α by inactivation of cGMP‐mediated CREB phosphorylation, leading to mitochondrial respiratory functional impairment concomitant with downregulation of glycolytic process. (B) Cropped western blot images of PGC‐1α and VDAC protein levels in the muscle of *PolgA* offspring at 9 months old with/without M‐HFD challenge (GAPDH and β‐tubulin were used for normalization; *n* = 6/group). (C) CpG islands of Pgc1a promoter region. (D) Relative enrichment folds of H3K4me3 in the muscle of *PolgA* offspring at 9 months old with/without M‐HFD challenge (*n* = 6/group). (E and F) GO analysis of differentially abundant epigenetic modification‐related gene signatures showing the effect of M‐HFD (E) or M‐HFD with *PolgA* (F) (*n* = 4/group). (G) Cropped western blots of oxidative phosphorylation respiration markers in *PolgA* offspring muscle challenged with M‐HFD (GAPDH was used for normalization; *n* = 6/group). Mean ± SEM, and each dot represents one litter. *p < 0.05 and ***p < 0.001 in WT vs. *PolgA* ^mut^, and #*p* < 0.05 in CD vs. HFD by two‐sided *p* values by two‐way ANOVA followed by Tukey's test (B–D).

M‐HFD challenge during pregnancy deteriorated mitochondrial metabolic processes including glycolysis, coenzyme metabolism and ATP biosynthesis in *PolgA* offspring muscle (Figure 6A). Consistently, M‐HFD revealed a dramatic decrease in the expression of peroxisome proliferator‐activated receptor γ coactivator α (protein: PGC‐1α, gene: *Pgc1a*), known as a central regulator of mitochondrial biogenesis (Supporting Information S1: S8), and voltage‐dependent anion channel (VDAC, Figure 6B), located in the mitochondrial outer membrane, which plays an important role in transporting mitochondrial metabolites (Supporting Information S1: S9). Interestingly, based on transcriptomics analysis, M‐HFD dynamically affected histone methylation pathways in offspring muscle (Figures 6E and 4A,B). Consistently, using ChIP‐PCR, we found that M‐HFD decreased histone H3 lysine 4 trimethylation (H3K4me3), known to activate gene expression, in the *Pgc1a* promoter due to M‐HFD and *PolgA* (Figure 6C,D). The oxidative phosphorylation markers were also *PolgA* dependently downregulated (Figures 6F and S4C). Together, we demonstrated that maternal HFD and *PolgA* synergistically reduce mitochondrial biogenesis and function.

### 3.5 Maternal HFD Downregulates GABA_A_ Receptor Signalling and Impairs Neuromuscular Junction in Aged Offspring

**FIGURE 7:**
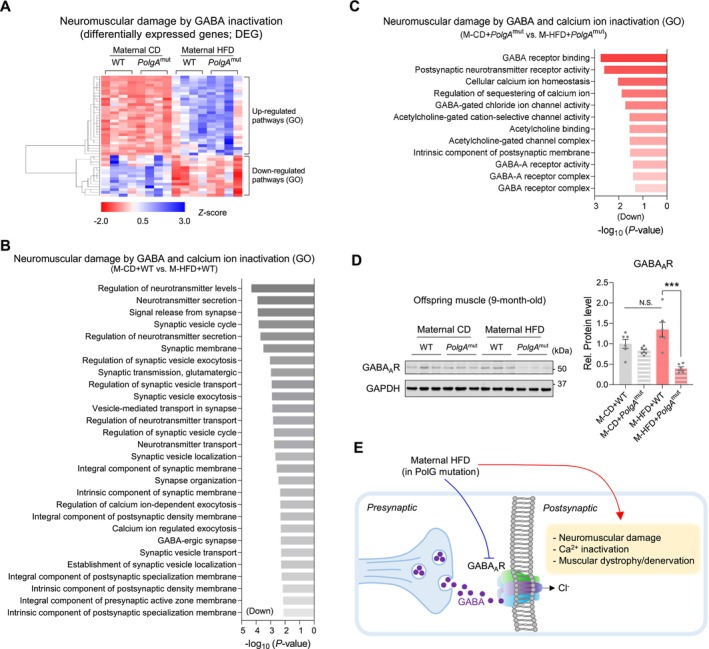
High‐calorie diet challenge during pregnancy induces neuromuscular damage by GABA_A_ receptor signalling inactivation in *PolgA* offspring muscle. (A–C) Heat map (A) and GO analyses of differentially enriched neuromuscular damage and calcium ion inactivation‐related genes in *PolgA* offspring muscle. Changes between M‐CD + WT and M‐HFD + WT (B) or M‐CD + *PolgA* ^mut^ and M‐HFD + *PolgA* ^mut^ (C) were compared (*n* = 4/group). (D) Cropped western blots of GABA_A_ receptor protein levels in *PolgA* muscle following M‐HFD challenge (GAPDH was used for normalization; *n* = 6/group). (E) Working model: GABA signalling inactivity‐mediated neuromuscular damage worsened by M‐HFD and *PolgA*. Mean ± SEM, and each dot represents one litter. ***p < 0.001 in WT vs. *PolgA* ^mut^ by two‐sided *p* values by two‐way ANOVA followed by Tukey's test (D).

Gamma‐aminobutyric acid (GABA) protects against age‐related loss of skeletal muscle mass and strength by promoting muscle protein synthesis (Supporting Information S1: S10). Consistently, our transcriptomic data show that M‐HFD suppressed pathways of GABA and calcium ions in the offspring muscle (Figure 7A,B). Of note, M‐HFD coupled with *PolgA* deteriorated the activities of calcium channels and neurotransmitter receptors, leading to dysfunction of postsynaptic regulation (Figures 7C and S5A). Consistently, M‐HFD reduced the activities of GABA_A_ receptors and acetylcholine binding channels, deteriorating postsynaptic neurotransmitter receptor activity (Figure 7C). GABA_A_ receptor protein levels were reduced in M‐HFD *PolgA* mice (Figure 7D). Our GO data also showed that M‐HFD downregulated the activity of G protein‐coupled receptors (GPCRs) and PI3/Akt pathways in the offspring muscle, especially in *PolgA* offspring (Figure S5B,C). Taken together, our data suggest that maternal HFD challenge during pregnancy markedly decreased GABA_A_ receptor‐related pathways, likely eliciting neuromuscular damage and denervation and offspring muscle atrophy (Figure 7E).

### 3.6 Maternal HFD Challenge Induces Apoptosis and Autophagy Process in PolG Mutator Offspring Muscle

Several studies have linked apoptosis to premature aging phenotypes in *PolgA* mice [28] (Supporting Information S1: S11 and S12). Consistently, it has been reported that PolG mutation elevates the autophagy process (Supporting Information S1: S13 and S14). Therefore, we next explored skeletal muscle apoptosis and autophagy processes in response to maternal HFD challenge in aged offspring mice, which were determined using RNA‐seq data of 9‐month‐old offspring muscle (Figure S6). We found that M‐HFD induced DNA damage, which might trigger intrinsic apoptotic responses (Figure S6A) (Supporting Information S1: S15). Moreover, consistent with the deleterious role of M‐HFD on muscle hypertrophy/atrophy signalling pathways (Figure 5), M‐HFD increased muscular autophagic responses (Figure S6A,B). Together, our data suggest that maternal HFD predisposes offspring muscle to apoptosis and autophagy, consistent with structural and functional impairment (Figures 2, 3, 4).

## 4 Discussion and Conclusion

In this study, we analysed the impacts of maternal HFD on the deterioration of offspring muscle structure and function. We demonstrated that maternal HFD not only deteriorates muscle structure and metabolism but also impairs muscle function including strength and exercise endurance (Supporting Information S1: S7). Maternal HFD resulted in intramuscular fibrosis. Interestingly, we found that GABA signalling was inactivated because of maternal HFD and *PolgA*, partially explaining the exercise intolerance in these mice. Our previous studies have shown that maternal HFD‐induced metabolic abnormality leads to mitochondrial dysfunction in offspring skeletal muscle [9, 10]; thus, the neuromuscular damage indicated by GABA down‐regulation might be due to impaired mitochondrial functions because the maintenance of GABA secretion and neural muscular function is energy‐demanding (Supporting Information S1: S16).

Fibrosis characterized by excessive collagen accumulation is not only a hallmark of muscular dystrophy but also a primary reason for the loss of strength and flexibility during aging [18], including adipose tissue (AT) and skeletal muscle (Supporting Information S1: S17 and S18). We previously showed that HFD‐induced obesity predisposes fibrosis in white AT (WAT) and skeletal muscle (Supporting Information S1: S17 and S18). In addition, MO increases the risk of fibrosis in the liver, which increases liver carcinogenesis (Supporting Information S1: S19). Similarly, we previously found that MO increases collagen accumulation in myocardium [21] and skeletal muscle [23], though the underlying mechanisms remain to be defined. In this study, we found that *PolgA* mice have elevated fibrosis in skeletal muscle, suggesting that such fibrosis is linked to mitochondrial dysfunction.

Mitochondria‐related diseases are devastating with no cure and no effective treatments [9] (Supporting Information S1: S20 and S21). The mutation rate of mtDNA is greater than that of nuclear DNA (Supporting Information S1: S22), and PolG is required for proofreading of mtDNA synthesis [27, 28]. Ablating PolG function results in dysfunction of mtDNA‐encoded subunits in complexes I, III, IV and V, leading to abnormal ATP production and other mitochondrial dysfunction (Supporting Information S1: S23). PolG‐related disorders have been recognized for their clinical implications, mainly showing muscle weakness, ataxia and peripheral neuropathy in all age populations (Supporting Information S1: S24). In particular, individuals with early‐onset diseases have the worst prognosis (Supporting Information S1: S24). Furthermore, mtDNA point mutations accumulate in various tissues during aging in humans, primates and rodents (Supporting Information S1: S25–S27). These increased mtDNA mutations reduce lifespan and lead to the premature onset of age‐related phenotypes (Supporting Information S1: S28). For this study, we utilized 6‐ and 9‐month‐old offspring mice. At 6 months of age, PolG mutated mice exhibited several progeroid phenotypes, such as loss of body fat, hair loss and spinal curvature by disruptions in proteins involved in energy metabolism and increased mitochondrial turnover (Supporting Information S1: S29 and S30). At 9 months old, the mutated mice showed severe signs of aging, including osteoporosis and sarcopenia (age‐dependent muscle loss). Importantly, the aging phenotypes profoundly accelerated in offspring of maternal HFD mice, demonstrating a strong developmental link between maternal HFD and the resulting premature aging of offspring, likely through accelerating mtDNA mutagenesis in offspring muscle. In agreement, we previously observed that obesity increases mtDNA heteroplasmy in oocytes (Supporting Information S1: S31).

In previous studies, we found that offspring skeletal muscle development is affected by MO and physical activity [9] (Supporting Information S1: S32). Particularly, the western diet, so called junk food, during pregnancy as well as lactation not only reduces muscle function and strength but also induces skeletal muscle atrophy in the offspring [26]. Here, we found that maternal HFD‐induced offspring muscle atrophy is linked to mitochondrial dysfunction, because atrophy was accelerated in *PolgA* mice. Additionally, we observed possible neuromuscular damage, which might also contribute to the atrophy (Supporting Information S1: S33 and S34). Furthermore, mitochondrial biogenesis is regulated by PGC‐1α, with its expression regulated by epigenetic modifications, including DNA and histone methylation [9]. The tri‐methylation of lysine 4 on histone H3 (H3K4me3), an epigenetic modification associated with gene activation (Supporting Information S1: S35), in the promoter of the *Pgc1a* gene was reduced because of maternal HFD and PolG mutation.

In conclusion, we show that maternal HFD challenge elicits premature aging of offspring skeletal muscle. Particularly, HFD during pregnancy impedes offspring muscle fitness, which might be due to intramuscular fibrosis and mitochondrial dysfunction concomitant with neuromuscular damage. Interestingly, the presence of PolG mutation worsens maternal HFD‐induced adverse changes in offspring muscle, suggesting mitochondrial impairment might have a key mediatory role. These data suggest that HFD intake during pregnancy has a substantial impact on the offspring muscle function and accelerates its aging. Our data suggested the mitochondria‐mediated metabolic dysfunction and atrophy in offspring muscle due to maternal HFD.

## Conflicts of Interest

The authors declare no conflicts of interest.

## Supporting information


**Figure S1.** In vivo metabolism in *PolgA* offspring muscle following M‐HFD. (A–C) Timeresolved respiratory exchange ratios (RERs) (A), fat oxidation (B), and CHO oxidation (C) of 9‐month‐old *PolgA* offspring born from mothers fed HFD during pregnancy (*n* = 6/group). Mean ± SEM, and each dot represents one litter. *p < 0.05 and **p < 0.01 in WT vs. *PolgA* ^mut^, and #*p* < 0.05 and ##*p* < 0.01 in CD vs. HFD by two‐sided *p* values by two‐way ANOVA followed by Tukey's test (A–C).
**Figure S2.** Secretory activity of offspring skeletal muscle following maternal HFD challenge. (A) heat map (left) and GO pathway analysis (right) of differentially enriched muscle hormonal secretion‐related gene signatures in the RNA sequencing of 9‐month‐old offspring muscle. Changes in muscle hormone activity between control diet and HFD were compared in a subset of wild‐type (WT) (*n* = 4/group). (B) Heat map (left) and GO pathway (right) of offspring muscle hormonal activity in *PolgA* following M‐HFD challenge (*n* = 4/group).
**Figure S3.** Skeletal muscle‐derived hormones in *PolgA* offspring muscle following MHFD. (A) Representative myokines in the RNA‐seq data of 9‐month‐old *PolgA* offspring muscle in response to M‐HFD challenge (*n* = 4/group). (B) Cropped western blots of FNDC5/irisin, APLN, BDNF, GDF11, PRDM16 and SPARC protein levels in 9‐month‐old *PolgA* offspring muscle in response to maternal HFD challenge during pregnancy (GAPDH and β‐tubulin were used for normalization; *n* = 6/group). (C) Pearson correlations between myokines and fitness capacity including maximal grip strength and total exercise time (*n* = 6/group). Mean ± SEM, and each dot represents one litter. *p < 0.05, **p < 0.01 and ***p < 0.001 in WT vs. *PolgA* ^mut^ by two‐sided *p* values by two‐way ANOVA followed by Tukey's test (B). *p* values are presented in each panel by Pearson correlation analysis (C).
**Figure S4.** Epigenetic regulations in *PolgA* offspring muscle in response to maternal HFD challenge. (A and B) GO analysis of differentially enriched epigenetic modification‐related gene signatures showing the effect of *PolgA* in either maternal control diet (A) or maternal HFD challenge (B) (*n* = 4/group). (C) Mean protein levels of mitochondrial oxidative phosphorylation markers in *PolgA* offspring muscle following M‐HFD challenge (*n* = 6/group). Mean ± SEM, and each dot represents one litter. *p < 0.05 and ***p < 0.001 in WT vs. *PolgA* ^mut^ by two‐sided *p* values by two‐way ANOVA followed by Tukey's test (C).
**Figure S5.** GO pathways in neuromuscular damages due to maternal HFD challenge and *PolgA*. (A) GO analysis of differentially enriched neuromuscular gene signatures in *PolgA* offspring muscle (*n* = 4/group). (B and C) GO analysis of differentially expressed downstream pathways of neuromuscular damages in response to M‐HFD (*n* = 4/group).
**Figure S6.** Maternal HFD induces skeletal muscle apoptosis and autophagy pathway signatures in *PolgA* offspring muscle. (A and B) GO pathways of apoptosis and autophagy in M‐HFD and *PolgA* offspring muscle (*n* = 4/group).

**Data S1.** Supporting information.

## Data Availability

The data that support the findings of this study are available from the corresponding author upon reasonable request.
