# Supplementary material for: High‐Calorie Diet During Pregnancy Leads to Muscular Fibrosis and Neuromuscular Damage in Offspring Mice
Source: J Cachexia Sarcopenia Muscle. 2025 Sep 16;16(5):e70027. doi: 10.1002/jcsm.70027 (PMC12440564; doi:10.1002/jcsm.70027)
Supplement: Supplementary file 1 — Figure S1. In vivo metabolism in PolgA offspring muscle following M‐HFD. (A–C) Timeresolved respiratory exchange ratios (RERs) (A), fat oxidation (B), and CHO oxidation (C) of 9‐month‐old PolgA offspring born from mothers fed HFD during pregnancy (n = 6/group). Mean ± SEM, and each dot represents one litter. *p < 0.05 and **p < 0.01 in WT vs. PolgA mut, and #p < 0.05 and ##p < 0.01 in CD vs. HFD by two‐sided p values by two‐way ANOVA followed by Tukey's test (A–C). Figure S2. Secretory activity of offspring skeletal muscle following maternal HFD challenge. (A) heat map (left) and GO pathway analysis (right) of differentially enriched muscle hormonal secretion‐related gene signatures in the RNA sequencing of 9‐month‐old offspring muscle. Changes in muscle hormone activity between control diet and HFD were compared in a subset of wild‐type (WT) (n = 4/group). (B) Heat map (left) and GO pathway (right) of offspring muscle hormonal activity in PolgA following M‐HFD challenge (n = 4/group). Figure S3. Skeletal muscle‐derived hormones in PolgA offspring muscle following MHFD. (A) Representative myokines in the RNA‐seq data of 9‐month‐old PolgA offspring muscle in response to M‐HFD challenge (n = 4/group). (B) Cropped western blots of FNDC5/irisin, APLN, BDNF, GDF11, PRDM16 and SPARC protein levels in 9‐month‐old PolgA offspring muscle in response to maternal HFD challenge during pregnancy (GAPDH and β‐tubulin were used for normalization; n = 6/group). (C) Pearson correlations between myokines and fitness capacity including maximal grip strength and total exercise time (n = 6/group). Mean ± SEM, and each dot represents one litter. *p < 0.05, **p < 0.01 and ***p < 0.001 in WT vs. PolgA mut by two‐sided p values by two‐way ANOVA followed by Tukey's test (B). p values are presented in each panel by Pearson correlation analysis (C). Figure S4. Epigenetic regulations in PolgA offspring muscle in response to maternal HFD challenge. (A and B) GO analysis of differentially enriche [file JCSM-16-e70027-s001.pdf]

Supplementary materials

High-calorie diet during pregnancy leads to muscular fibrosis and neuromuscular damage in offspring mice

Jun Seok Son, Song Ah Chae, Yoon Ha Chun, Hongyang Wang, Zhihua Jiang, Min Du

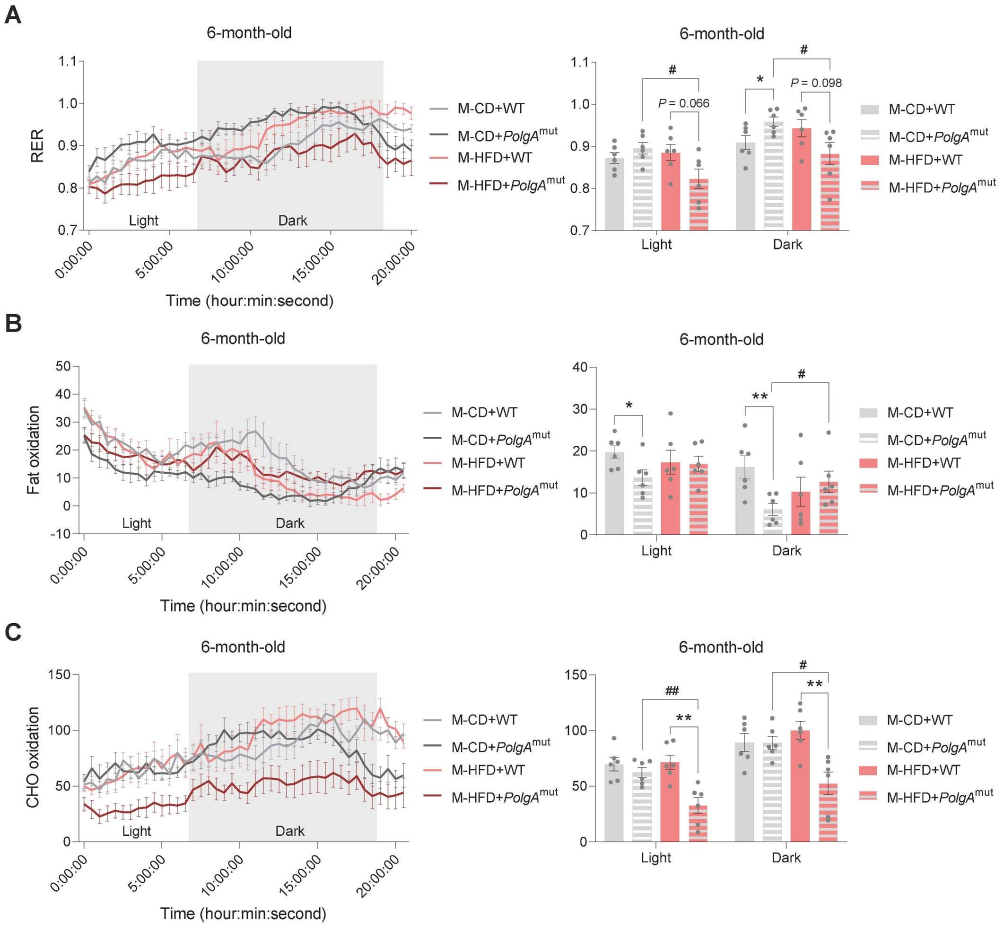

Supplementary Fig. 1. In vivo metabolism in *PolgA* offspring muscle following M-HFD. A-C, Time-resolved respiratory exchange ratios (RERs) (A), fat oxidation (B), and CHO oxidation (C) of 9-month-old *PolgA* offspring born from mothers fed HFD during pregnancy (n = 6/group). Mean ± s.e.m., and each dot represents one litter. $^{*}P < 0.05$ and $^{**}P < 0.01$ in WT vs. *PolgA*^{mut}, and $^{\#}P < 0.05$ and $^{\#\#}P < 0.01$ in CD vs. HFD by two-sided P values by two-way ANOVA followed by Tukey's test (A-C).

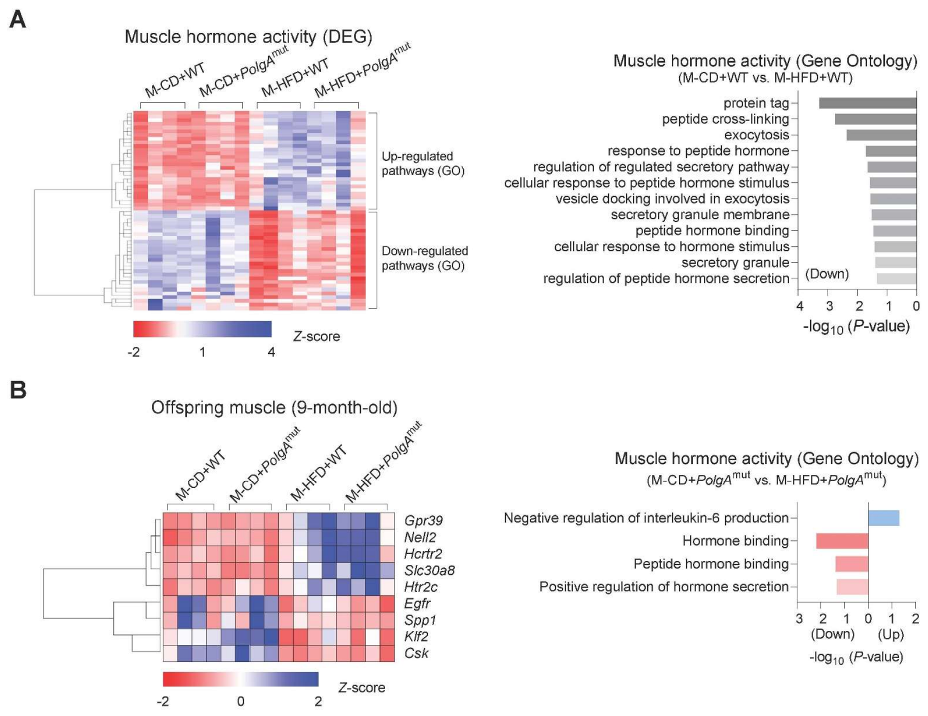

Supplementary Fig. 2. Secretory activity of offspring skeletal muscle following maternal HFD challenge. A, heat map (left) and GO pathway analysis (right) of differentially enriched muscle hormonal secretion-related gene signatures in the RNA sequencing of 9-month-old offspring muscle. Changes in muscle hormone activity between control diet and HFD were compared in a subset of wild-type (WT) (n = 4/group). **B,** Heat map (left) and GO pathway (right) of offspring muscle hormonal activity in *PolgA* following M-HFD challenge (n = 4/group).

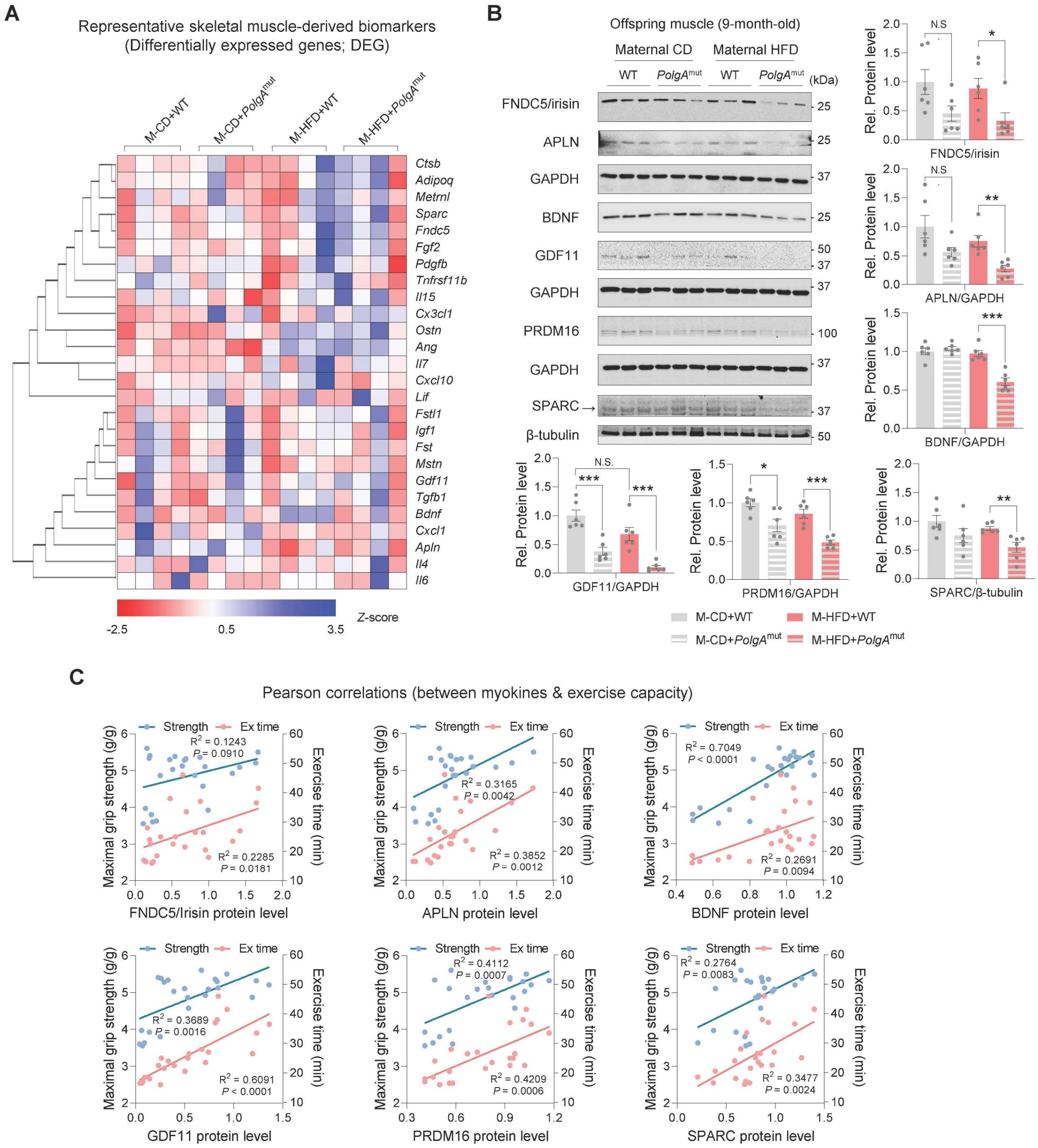

Supplementary Fig. 3. Skeletal muscle-derived hormones in *PolgA* offspring muscle following M-HFD. A, Representative myokines in the RNA-seq data of 9-month-old *PolgA* offspring muscle in response to M-HFD challenge (n = 4/group). **B,** Cropped western blots of FNDC5/irisin, APLN, BDNF, GDF11, PRDM16, and SPARC protein levels in 9-month-old *PolgA* offspring muscle in response to maternal HFD challenge during pregnancy (GAPDH and β-tubulin were used for normalization; n =

6/group). **C**, Pearson correlations between myokines and fitness capacity including maximal grip strength and total exercise time (n = 6/group). Mean ± s.e.m., and each dot represents one litter. **P* < 0.05, ***P* < 0.01, and ****P* < 0.001 in WT vs. *PolgA*^{mut} by two-sided *P* values by two-way ANOVA followed by Tukey's test (B). *P* values are presented in each panel by Pearson correlation analysis (C).

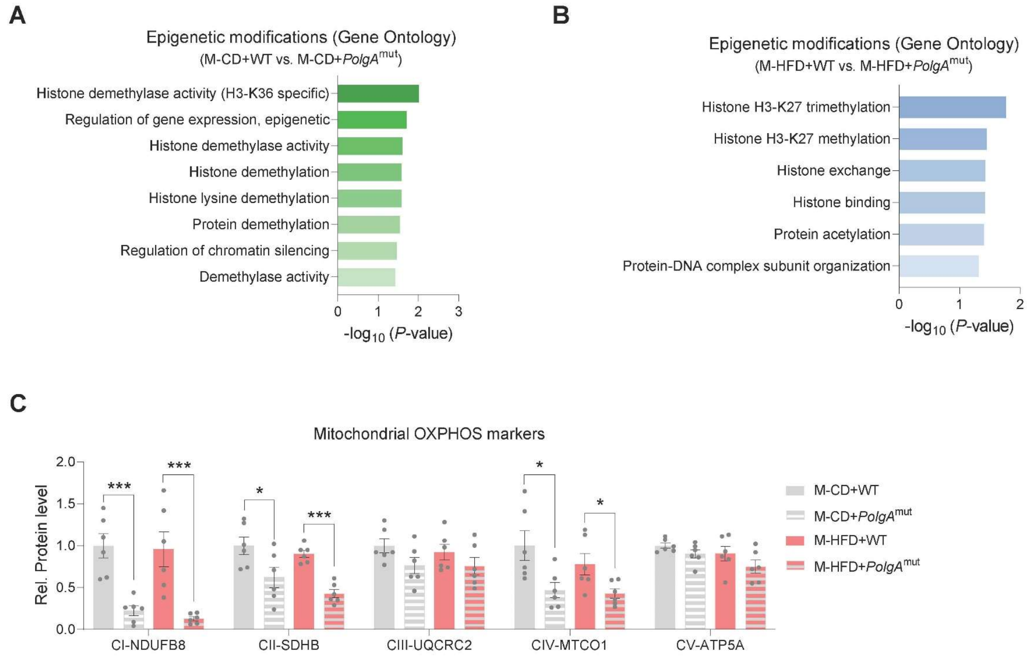

Supplementary Fig. 4. Epigenetic regulations in *PolgA* offspring muscle in response to maternal HFD challenge. A and **B,** GO analysis of differentially enriched epigenetic modification-related gene signatures showing the effect of *PolgA* in either maternal control diet (A) or maternal HFD challenge (B) (n = 4/group). **C,** Mean protein levels of mitochondrial oxidative phosphorylation markers in *PolgA* offspring muscle following M-HFD challenge (n = 6/group). Mean ± s.e.m., and each dot represents one litter. *$P < 0.05$ and ***$P < 0.001$ in WT vs. $PolgA^{mut}$ by two-sided P values by two-way ANOVA followed by Tukey's test (C).

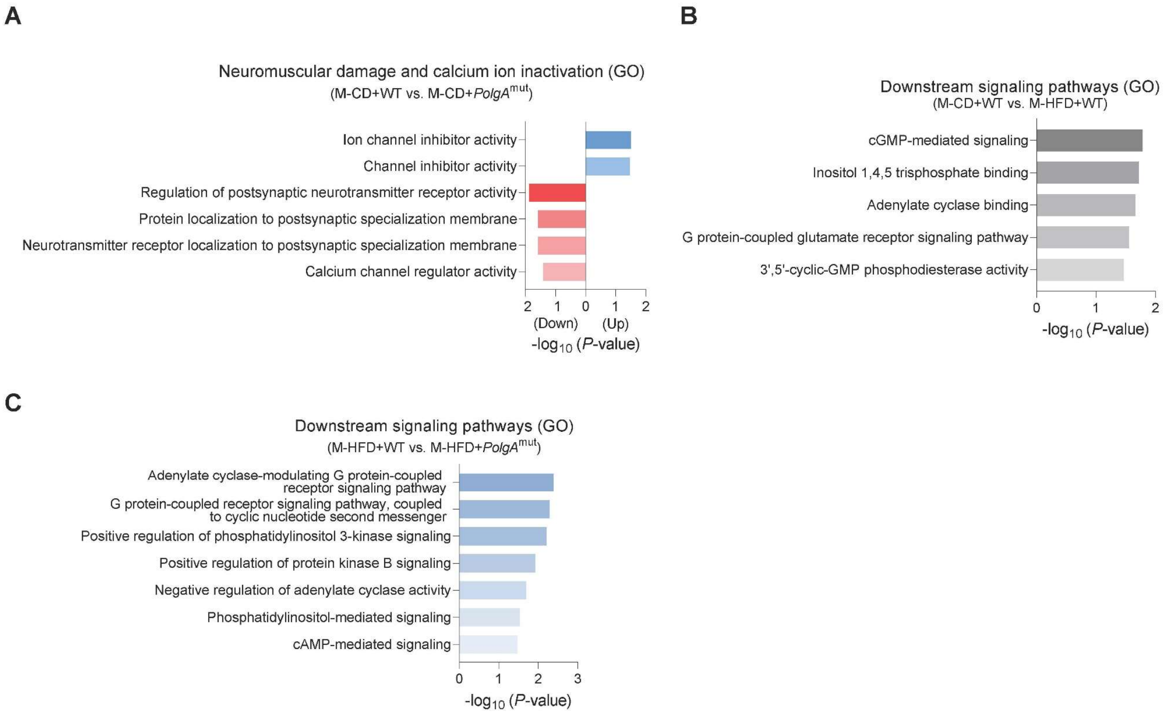

Supplementary Fig. 5. GO pathways in neuromuscular damages due to maternal HFD challenge and *PolgA*. A, GO analysis of differentially enriched neuromuscular gene signatures in *PolgA* offspring muscle (n = 4/group). **B** and **C**, GO analysis of differentially expressed downstream pathways of neuromuscular damages in response to M-HFD (n = 4/group).

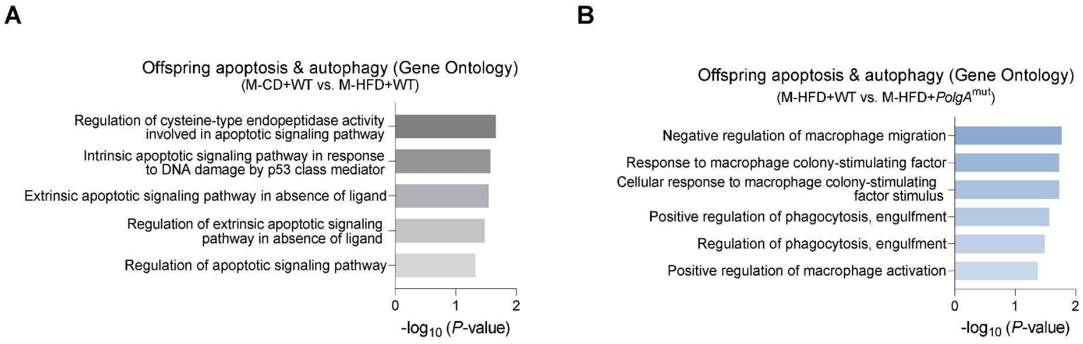

Supplementary Fig. 6. Maternal HFD induces skeletal muscle apoptosis and autophagy pathway signatures in *PolgA* offspring muscle. A and **B**, GO pathways of apoptosis and autophagy in M-HFD and *PolgA* offspring muscle (n = 4/group).

References

S1. Rao VV, Mohanty A. Immunohistochemical Identification of Muscle Fiber Types in Mice Tibialis Anterior Sections. Bio Protoc. 2019;9:e3400. doi:10.21769/BioProtoc.3400

S2. Pinel S, Kelp NY, Bugeja JM, Bolsterlee B, Hug F, Dick TJM. Quantity versus quality: Age-related differences in muscle volume, intramuscular fat, and mechanical properties in the triceps surae. Exp Gerontol. 2021;156:111594. doi:10.1016/j.exger.2021.111594

S3. Bodine SC, Baehr LM. Skeletal muscle atrophy and the E3 ubiquitin ligases MuRF1 and MAFbx/atrogin-1. American journal of physiology Endocrinology and metabolism. 2014;307:E469-84. doi:10.1152/ajpendo.00204.2014

S4. Murton AJ, Constantin D, Greenhaff PL. The involvement of the ubiquitin proteasome system in human skeletal muscle remodelling and atrophy. Biochim Biophys Acta. 2008;1782:730-43. doi:10.1016/j.bbadis.2008.10.011

S5. Chow LS, Gerszten RE, Taylor JM, Pedersen BK, van Praag H, Trappe S, et al. Exerkines in health, resilience and disease. Nature reviews Endocrinology. 2022;18:273-89. doi:10.1038/s41574-022-00641-2

S6. Hoffmann C, Weigert C. Skeletal Muscle as an Endocrine Organ: The Role of Myokines in Exercise Adaptations. Cold Spring Harbor perspectives in medicine. 2017;7:doi:10.1101/cshperspect.a029793

S7. Pedersen BK, Febbraio MA. Muscles, exercise and obesity: skeletal muscle as a secretory organ. Nature reviews Endocrinology. 2012;8:457-65. doi:10.1038/nrendo.2012.49

S8. LeBleu VS, O'Connell JT, Gonzalez Herrera KN, Wikman H, Pantel K, Haigis MC, et al. PGC-1α mediates mitochondrial biogenesis and oxidative phosphorylation in cancer cells to promote metastasis. Nature cell biology. 2014;16:992-1003, 1-15. doi:10.1038/ncb3039

S9. Shoshan-Barmatz V, De Pinto V, Zweckstetter M, Raviv Z, Keinan N, Arbel N. VDAC, a multi-functional mitochondrial protein regulating cell life and death. Molecular aspects of medicine. 2010;31:227-85. doi:10.1016/j.mam.2010.03.002

S10. Jin H, Oh HJ, Lee BY. GABA Prevents Age-Related Sarcopenic Obesity in Mice with High-Fat-Diet-Induced Obesity. Cells. 2023;12:doi:10.3390/cells12172146

S11. Zhang D, Mott JL, Farrar P, Ryerse JS, Chang SW, Stevens M, et al. Mitochondrial DNA mutations activate the mitochondrial apoptotic pathway and cause dilated cardiomyopathy. Cardiovascular research. 2003;57:147-57. doi:10.1016/s0008-6363(02)00695-8

S12. Kujoth GC, Bradshaw PC, Haroon S, Prolla TA. The role of mitochondrial DNA mutations in mammalian aging. PLoS genetics. 2007;3:e24. doi:10.1371/journal.pgen.0030024

S13. Medeiros TC, Thomas RL, Ghillebert R, Graef M. Autophagy balances mtDNA synthesis and degradation by DNA polymerase POLG during starvation. The Journal of cell biology. 2018;217:1601-11. doi:10.1083/jcb.201801168

S14. Li-Harms X, Milasta S, Lynch J, Wright C, Joshi A, Iyengar R, et al. Mito-protective autophagy is impaired in erythroid cells of aged mtDNA-mutator mice. Blood. 2015;125:162-74. doi:10.1182/blood-2014-07-586396

S15. Roos WP, Kaina B. DNA damage-induced cell death by apoptosis. Trends in molecular medicine. 2006;12:440-50. doi:10.1016/j.molmed.2006.07.007

S16. Moujalled D, Strasser A, Liddell JR. Molecular mechanisms of cell death in neurological diseases. Cell death and differentiation. 2021;28:2029-44. doi:10.1038/s41418-021-00814-y

S17. Liu X, Zhao L, Chen Y, Gao Y, Tian Q, Son JS, et al. Obesity induces adipose fibrosis and collagen cross-linking through suppressing AMPK and enhancing lysyl oxidase expression. Biochimica et biophysica acta Molecular basis of disease. 2022;1868:166454. doi:10.1016/j.bbadis.2022.166454

S18. Liu X, Zhao L, Gao Y, Chen Y, Tian Q, Son JS, et al. AMP-activated protein kinase inhibition in fibro-adipogenic progenitors impairs muscle regeneration and increases fibrosis. Journal of cachexia, sarcopenia and muscle. 2022;doi:10.1002/jcsm.13150

S19. Moeckli B, Delaune V, Gilbert B, Peloso A, Oldani G, El Hajji S, et al. Maternal obesity increases the risk of hepatocellular carcinoma through the transmission of an altered gut microbiome. JHEP reports : innovation in hepatology. 2024;6:101056. doi:10.1016/j.jhepr.2024.101056

S20. Tian C, Liu Y, Li Z, Zhu P, Zhao M. Mitochondria Related Cell Death Modalities and Disease. Front Cell Dev Biol. 2022;10:832356. doi:10.3389/fcell.2022.832356

S21. Molnar MJ, Kovacs GG. Mitochondrial diseases. Handbook of clinical neurology. 2017;145:147-55. doi:10.1016/b978-0-12-802395-2.00010-9

S22. Ballard JW, Whitlock MC. The incomplete natural history of mitochondria. Molecular ecology. 2004;13:729-44. doi:10.1046/j.1365-294x.2003.02063.x

S23. Wallace DC, Fan W. The pathophysiology of mitochondrial disease as modeled in the mouse. Genes Dev. 2009;23:1714-36. doi:10.1101/gad.1784909
S24. Cohen BH, Chinnery PF, Copeland WC. POLG-Related Disorders. In: Adam MP, Feldman J, Mirzaa GM, Pagon RA, Wallace SE, Bean LJH, et al. editors. Seattle (WA): University of Washington, Seattle
Copyright © 1993-2024, University of Washington, Seattle. GeneReviews is a registered trademark of the University of Washington, Seattle. All rights reserved.; 1993.
S25. Corral-Debrinski M, Horton T, Lott MT, Shoffner JM, Beal MF, Wallace DC. Mitochondrial DNA deletions in human brain: regional variability and increase with advanced age. Nat Genet. 1992;2:324-9. doi:10.1038/ng1292-324
S26. Schwarze SR, Lee CM, Chung SS, Roecker EB, Weindruch R, Aiken JM. High levels of mitochondrial DNA deletions in skeletal muscle of old rhesus monkeys. Mechanisms of ageing and development. 1995;83:91-101. doi:10.1016/0047-6374(95)01611-3
S27. Khaidakov M, Heflich RH, Manjanatha MG, Myers MB, Aidoo A. Accumulation of point mutations in mitochondrial DNA of aging mice. Mutation research. 2003;526:1-7. doi:10.1016/s0027-5107(03)00010-1
S28. Trifunovic A, Wredenberg A, Falkenberg M, Spelbrink JN, Rovio AT, Bruder CE, et al. Premature ageing in mice expressing defective mitochondrial DNA polymerase. Nature. 2004;429:417-23. doi:10.1038/nature02517
S29. Trease AJ, Totusek S, Lichter EZ, Stauch KL, Fox HS. Mitochondrial DNA Instability Supersedes Parkin Mutations in Driving Mitochondrial Proteomic Alterations and Functional Deficits in Polg Mutator Mice. International journal of molecular sciences. 2024;25:doi:10.3390/ijms25126441
S30. Woodall BP, Orogo AM, Najor RH, Cortez MQ, Moreno ER, Wang H, et al. Parkin does not prevent accelerated cardiac aging in mitochondrial DNA mutator mice. JCI insight. 2019;5:doi:10.1172/jci.insight.127713
S31. Chen Y, Ma G, Gai Y, Yang Q, Liu X, de Avila JM, et al. AMPK Suppression Due to Obesity Drives Oocyte mtDNA Heteroplasmy via ATF5-POLG Axis. Advanced science (Weinheim, Baden-Wurttemberg, Germany). 2024;e2307480. doi:10.1002/advs.202307480
S32. Zhao L, Law NC, Gomez NA, Son J, Gao Y, Liu X, et al. Obesity Impairs Embryonic Myogenesis by Enhancing BMP Signaling within the Dermomyotome. Advanced science (Weinheim, Baden-Wurttemberg, Germany). 2021;8:e2102157. doi:10.1002/advs.202102157
S33. Kubat GB, Bouhamida E, Ulger O, Turkel I, Pedriali G, Ramaccini D, et al. Mitochondrial dysfunction and skeletal muscle atrophy: Causes, mechanisms, and treatment strategies. Mitochondrion. 2023;72:33-58. doi:10.1016/j.mito.2023.07.003
S34. Langer HT, Senden JMG, Gijsen AP, Kempa S, van Loon LJC, Spuler S. Muscle Atrophy Due to Nerve Damage Is Accompanied by Elevated Myofibrillar Protein Synthesis Rates. Frontiers in physiology. 2018;9:1220. doi:10.3389/fphys.2018.01220
S35. Wang H, Fan Z, Shliaha PV, Miele M, Hendrickson RC, Jiang X, et al. H3K4me3 regulates RNA polymerase II promoter-proximal pause-release. Nature. 2023;615:339-48. doi:10.1038/s41586-023-05780-8
