# Supplementary material for: High‐Calorie Diet During Pregnancy Leads to Muscular Fibrosis and Neuromuscular Damage in Offspring Mice
Source: J Cachexia Sarcopenia Muscle. 2025 Sep 16;16(5):e70027. doi: 10.1002/jcsm.70027 (PMC12440564; doi:10.1002/jcsm.70027)

Uncropped western blot images

Figure 4D (sample 1-3)

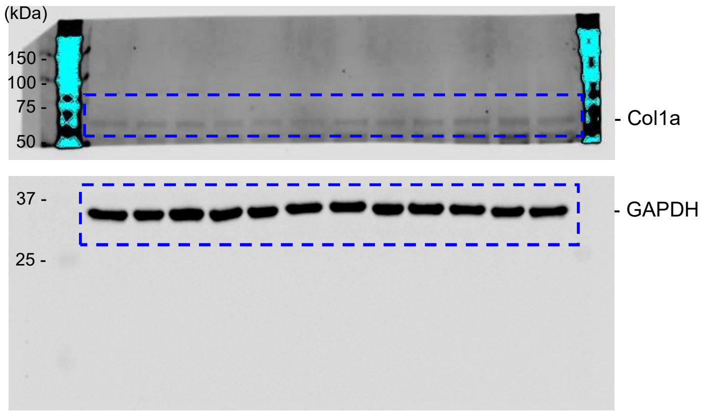

Figure 4D (sample 4-6)

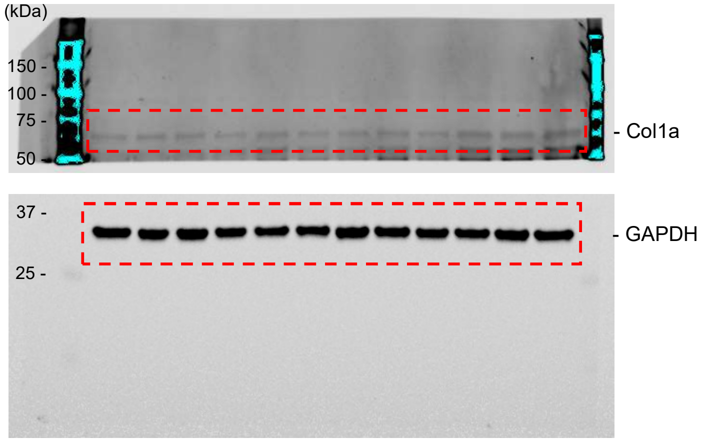

Figure 4E (sample 1-3)

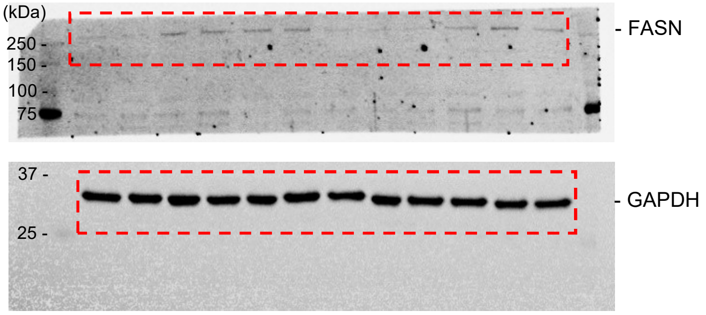

Figure 4E (sample 4-6)

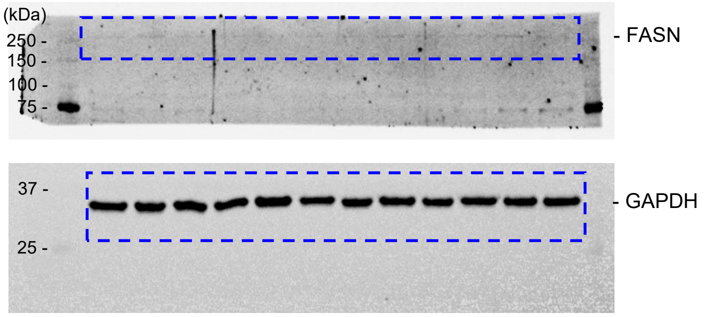

Figure 4E (sample 1-3)

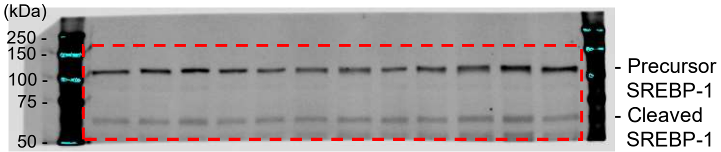

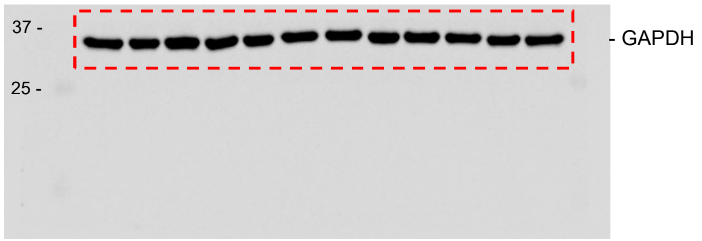

Figure 4E (sample 4-6)

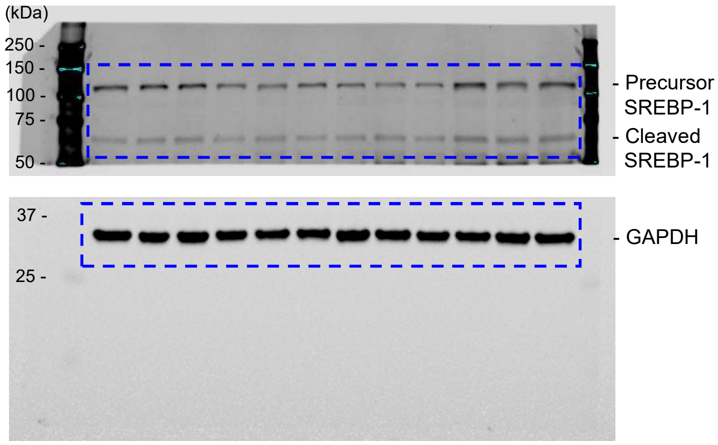

Figure 5D (sample 1-3)

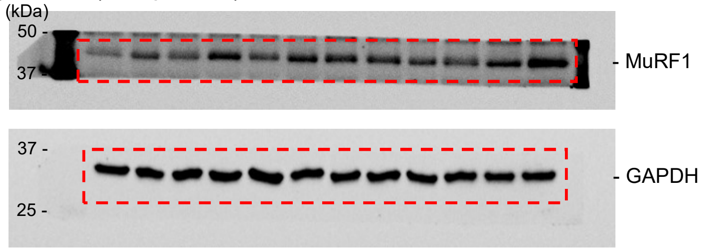

Figure 5D (sample 4-6)

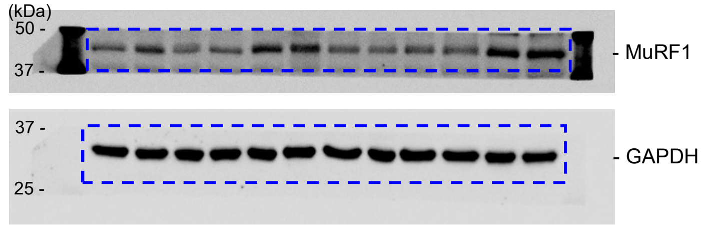

Figure 5D (sample 1-3)

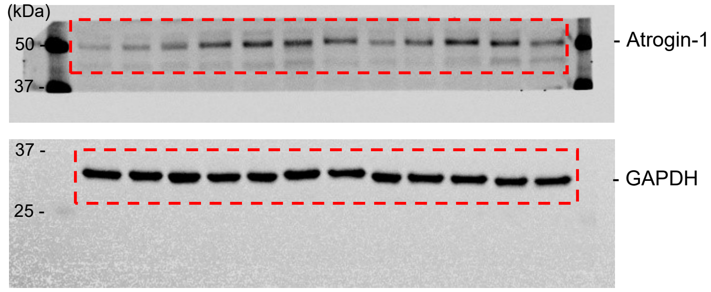

Figure 5D (sample 4-6)

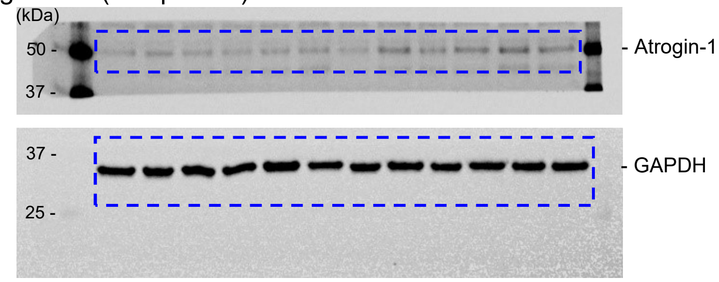

Figure 5E (sample 1-3)

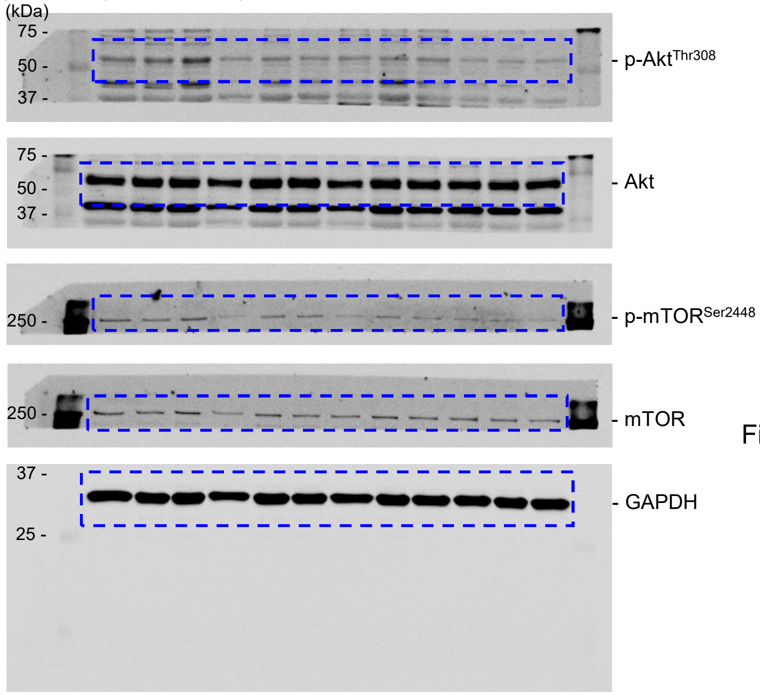

Figure 5E (sample 4-6)

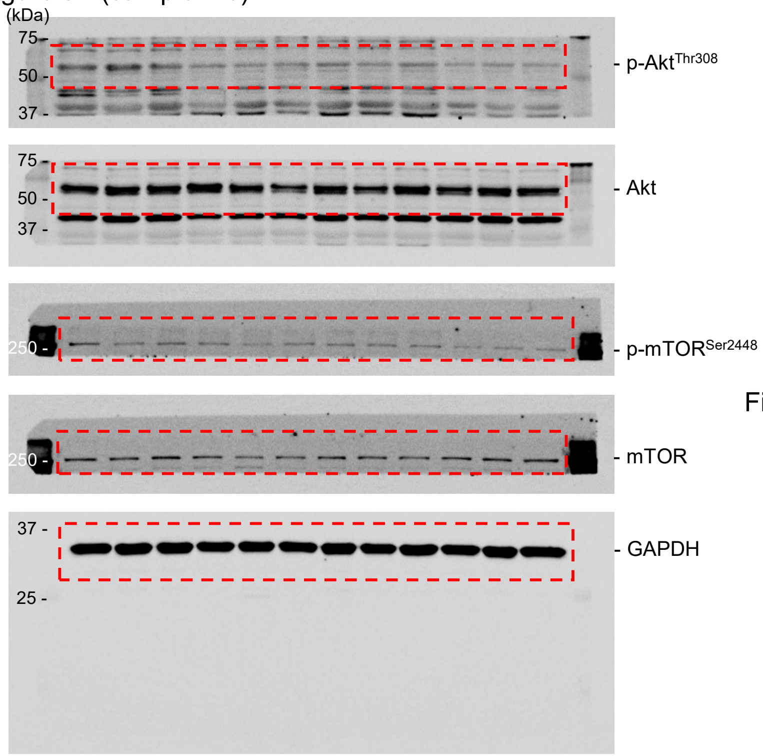

Figure 5E (sample 1-3)

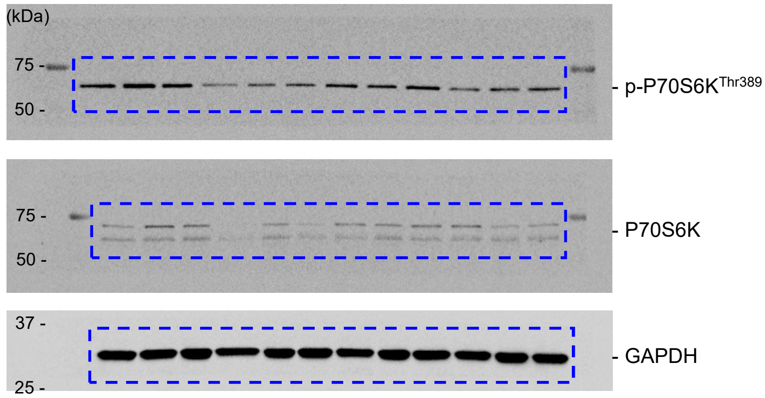

Figure 5E (sample 4-6)

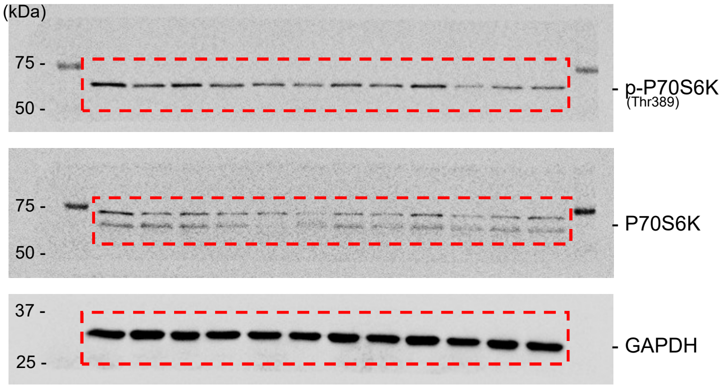

Figure 6B (sample 1-3)

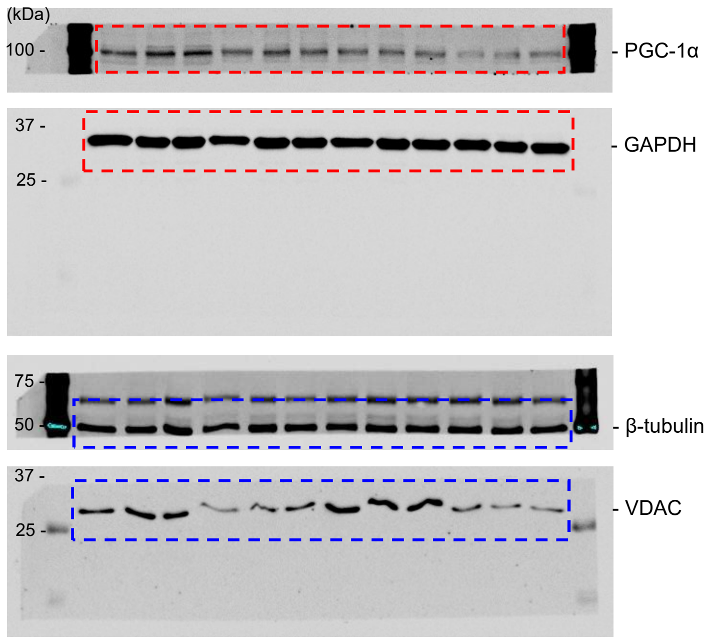

Figure 6B (sample 4-6)

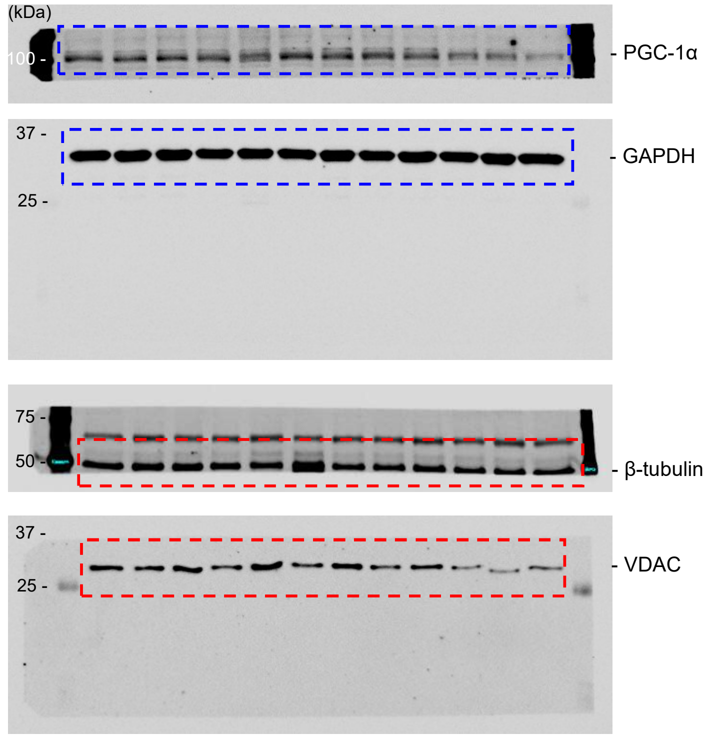

Figure 7D (sample 1-3)

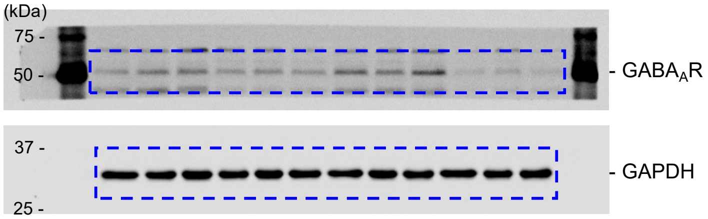

Figure 7D (sample 4-6)

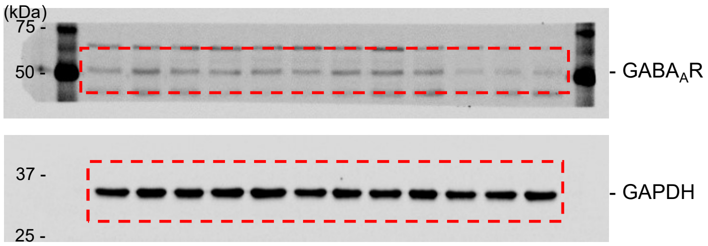

Supplementary Figure 3B (sample 1-3)

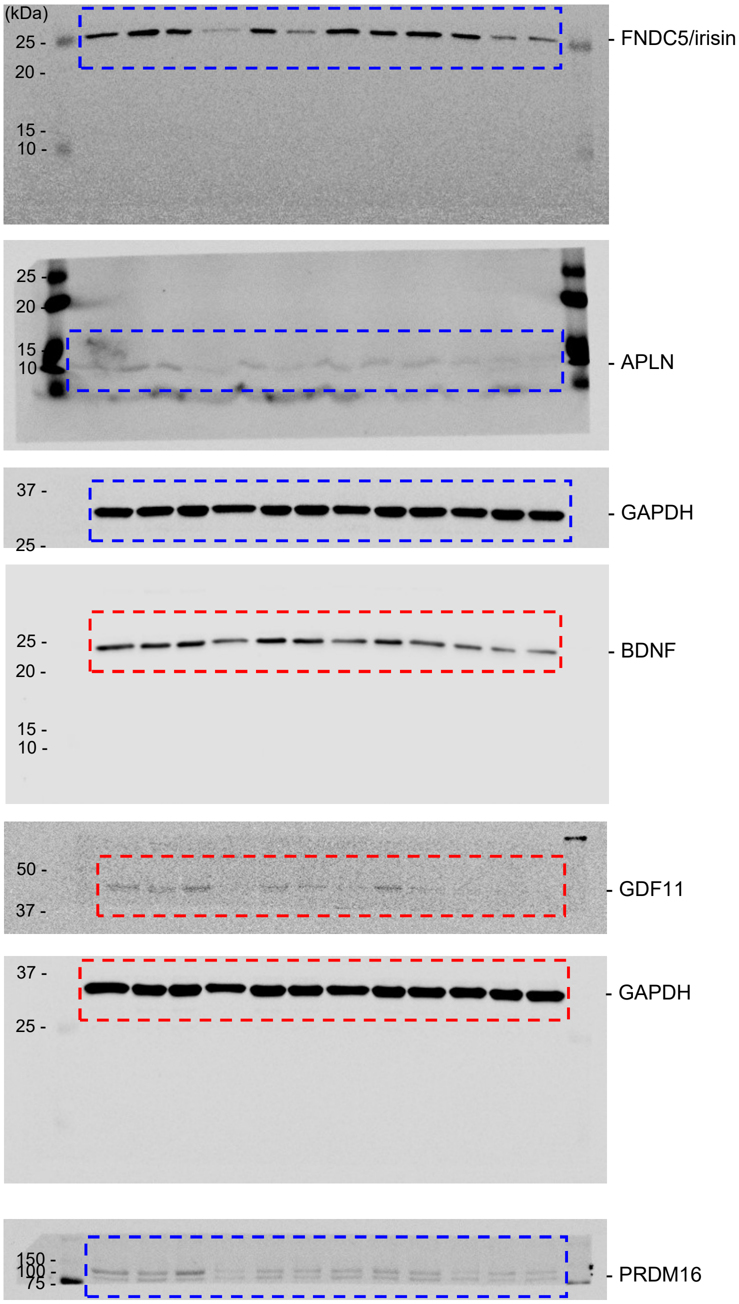

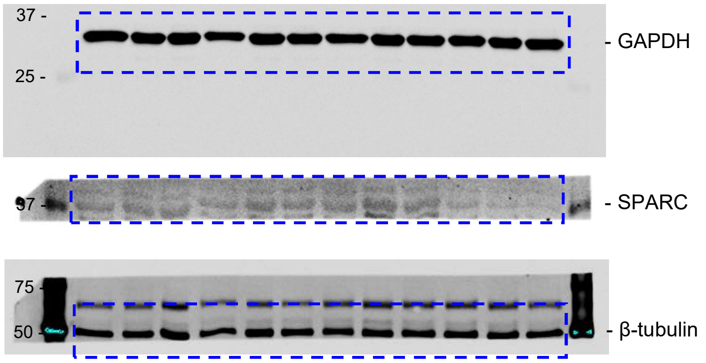

Supplementary Figure 3B (sample 1-3)

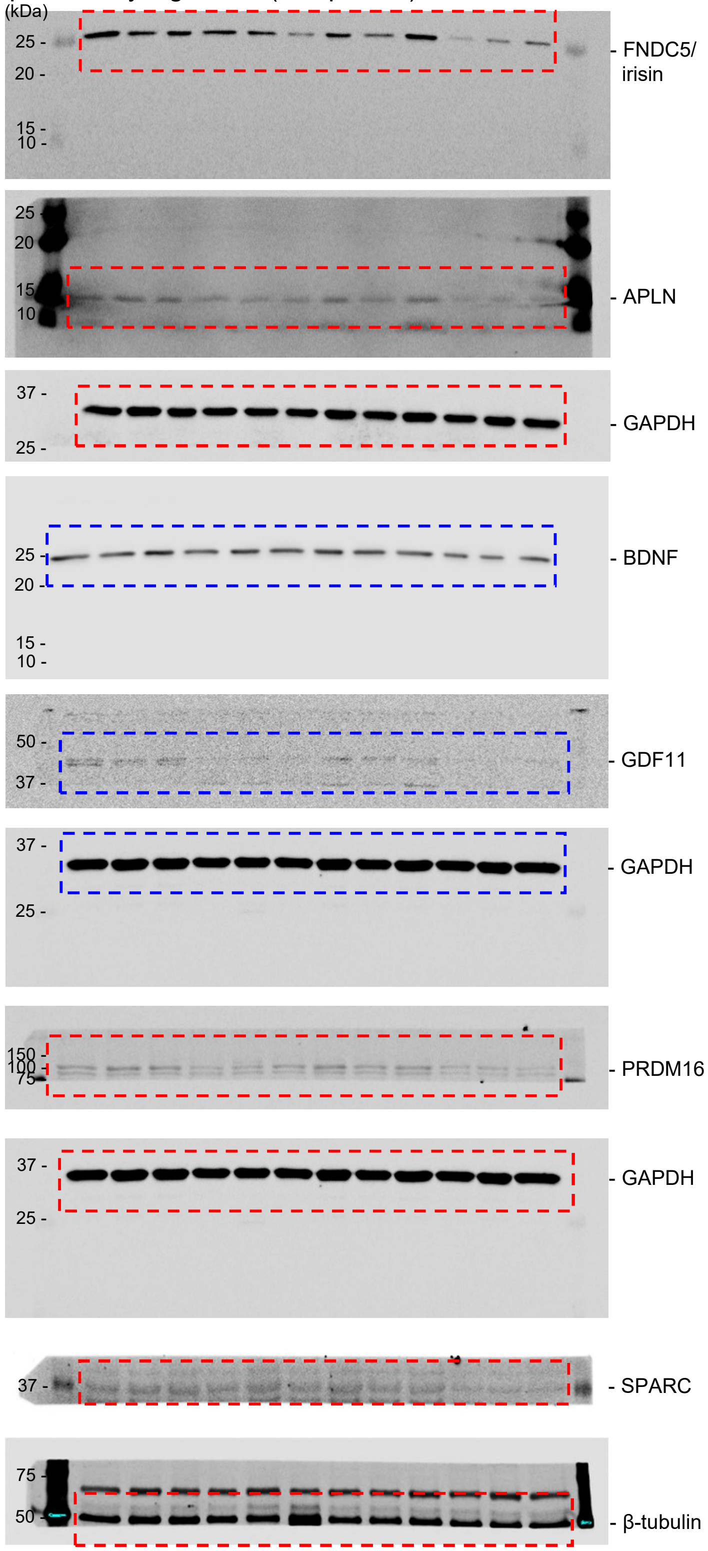

Supplement: Supplementary file 2 — Data S1. Supporting information. [file JCSM-16-e70027-s002.pdf]
